# Who Rules? Support Coalitions and Regime Survival, 1789–2020

**DOI:** 10.1177/00104140251369338

**Published:** 2025-08-20

**Authors:** Carl Henrik Knutsen, Sirianne Dahlum, Magnus Bergli Rasmussen, Tore Wig

**Affiliations:** 1 University of Oslo, Oslo, Norway; 2 University of South-Eastern Norway, Drammen, Norway

**Keywords:** democratization and regime change, non-democratic regimes, political regimes

## Abstract

All political regimes rely on coalitions of supporters to govern and remain in power. We discuss, specify, and test arguments pertaining to how the numerical size as well as social diversity of regime support coalitions influence regime longevity. We use recently collected data on support coalition characteristics, with global coverage and time series exceeding 230 years, and find that larger as well as more diverse regime support coalitions are positively related to regime longevity. Both patterns hold up in different time periods and in sub-samples including only autocratic regimes. We also discuss and find indications that increased coalition size may limit certain types of regime breakdown (e.g., from popular uprisings) but not others (e.g., coups). Coalition diversity is negatively related to different types of regime breakdown, and these patterns may reflect that diverse coalitions offer regimes access to variegated power resources that enable them to mitigate different threats.

## Introduction

Why are some political regimes more durable than others? The survival and breakdown of regimes – both democratic and autocratic ones – is often explained with reference to political institutions. Scholars studying autocracies have, for example, highlighted how autocratic elections influence regime survival (Gandhi & Lust-Okar, 2009) and, for democracies, prominent arguments point to destabilizing effects of presidentialism (Linz, 1990). Yet, institutions are not the only salient regime features that influence durability. Characteristics of political actors, and especially empowered regime supporters “behind the throne”, may also matter. In this paper, we assess the role of *regime support coalitions*, understood as the coalition of individuals that support the current regime, and whose support substantially strengthens the regime’s hold on power. More specifically, we theorize and empirically assess how the size and social diversity of regime coalitions relate to regime breakdown. To briefly preview our main results, we find that larger as well as more diverse coalitions correspond with more durable regimes.

Whether inclusive or diverse coalitions mitigates (e.g., Lijphart, 1999; Powell, 2000) or exacerbates (e.g., Horowitz, 2000; Huntington, 1968) various sources or types of political instability has been a core topic of debate in political science for decades. For instance, inclusive and diverse coalitions have been argued to mitigate social tension, improve policy performance through cooperation, and avoid leaving key groups as potential “spoilers” with incentives to contest the regime (e.g., Lijphart, 1969, 1999). On the other hand, inclusive and diverse coalitions have also been argued to lead to destabilizing gridlock and inability to take decisive action (e.g., Huntington, 1968), or, when it comes to diversity – at least within specific institutional configurations – further segmentation and intensification of inter-group conflict over time as well as incentives for larger groups to alter the system (Horowitz, 2000). Although many contributions have focused on more specific groups and notions of diversity (such as language, religion, or ethnicity) and particular regimes (notably democracies), our arguments and empirical tests, which consider different forms of diversity and pertain to variation across autocratic as well as democratic regimes, still speak to these classic debates.

For our empirical tests, we use recently collected data that contain direct, expert-coded measures of size and social composition of regime support coalitions, with time series exceeding 230 years.^1^ Over this time span, regime support coalitions have generally become broader and more diverse. These trends in our data mirror the more qualitative-historical descriptions of a rich literature addressing such socio-political (and related, underlying economic) developments and their further consequences for regime stability and change. One prominent strand links the emergence and collaboration of various social groups to political revolutions (e.g., Moore, 1966; Skocpol, 1979). Another strand views the rise of new – especially urban – groups as part of a broader complex of “modernization”, facilitating democratizing regime changes in the 19th and 20th centuries (Lipset, 1959). Yet other seminal contributions link the emergence and political inclusion of social groups to disorder and instability, more generally (e.g., Huntington, 1968). These contributions notwithstanding, exactly how a widening or diversification of support coalitions relate to regime stability and change remains unresolved empirical questions. And, despite the above-described broad trends, regimes have always varied – and still vary – extensively in how sizeable and heterogeneous their coalitions of core supporters are.^2^ We theorize and test how such variation in regime support coalition composition influences the resiliency of both democratic and autocratic regimes to different types of threats, and thus regime survival.

The first coalition aspect that we consider is numerical size. Different notions of coalition size, pertaining for instance to the number of supporters backing a particular leader *or* the wider regime,^3^ figure prominently also in more recent theoretical work on leader- or regime survival. Large coalitions could incentivize leaders and other policy makers to pursue policies that benefit larger population segments, thereby mitigating risks of mass revolutions or rebellion that may put the (leader and the wider) regime at risk (see, e.g., Bueno De Mesquita, 2010; Bueno de Mesquita & Smith, 2009). Yet, large coalitions could simultaneously increase threats to (leader and) regime survival that come from within the ruling elite, through increased internal competition and fractionalization, thereby enhancing coup risks (Roessler, 2011; Svolik, 2009). Hence, the overall relationship between coalition size and regime duration must be assessed empirically. However, implications of arguments linking coalition size to regime survival have been hard to test directly, absent measures of coalition size with extensive cross-national coverage. Existing large-n research has relied on proxies such as indicators from Polity capturing institutional constraints on the executive (Bueno de Mesquita et al., 2003), more specific institutional indicators from V-Dem (Bueno de Mesquita & Smith, 2022), or the Geddes et al. (2014) autocracy classification (Mattes et al., 2016). While such measures of institutions and regime types presumably correlate with coalition size, they also capture other aspects of political systems (Kennedy, 2009; Morrow, De Mesquita, Siverson and Smith, 2008) that may have independent effects on regime survival. Hence, we make an empirical contribution by testing how our more direct measure of coalition size relates to regime survival in a global sample.

The second coalition aspect that we consider is social diversity. When doing so, we build on existing arguments (e.g., Gallagher & Hanson, 2015; Slater, 2010) pertaining to the *heterogeneity in social characteristics* of coalition members and how this shapes the regime’s access to different types of power resources. We further specify and empirically assess this argument by using a measure of regime support coalition diversity, which is only moderately correlated with coalition size in our data (Pearson’s r = .33). As indicated, “power resources” is a key concept in our argument linking coalition diversity to regime survival, and it may be defined broadly as attributes or properties of social groups that enable them to reward or punish other actors (see Korpi, 2006, p. 172). We assume that regime elites can draw on the bundles of power resources held by the different social groups incorporated in their support coalitions (see also Slater, 2010), be it military officers, religious leaders, industrial workers, or farmers. Hence, the coalition’s social composition shapes the capacities of regime elites to deal with different threats. For instance, regimes having the state bureaucracy as support coalition partners may benefit from the increased monitoring and implementation capacity that follows from tight control over the state administration. Regimes supported by business elites may have easier access to financial resources that can be used to co-opt threats or invest in repressive capacity. Regimes supported by rural workers can draw on the latter group’s large numbers to mobilize large, countervailing pro-regime protests when faced with challenging anti-regime protests. Regimes that incorporate state bureaucrats, business elites, *and* rural workers in their coalitions can access *all* three types of resources. This offers the regime flexibility in its response to a particular threat, but also the ability to counter various types of threats by matching the nature of the threat with an effective response. More generally, we expect that more diverse support coalitions enhance regime survival, even when holding coalition size constant, since the regime can access a wider range of power resources.

Our measures of regime support coalition size and diversity are both based on questions designed by the authors and collected using the pool of country experts from the Varieties of Democracy (V-Dem) project. We combine these measures, which can vary also over time within a regime, with detailed data on regime identities and dates of breakdown (from Djuve et al., 2020). The resulting analyses cover more than 1500 regimes in about 170 polities, with time series from 1789–2020. In our benchmark, assuming monotonic relationships and considering all forms of regime breakdown, we find that both larger *and* more diverse coalitions, on average, are positively related to regime longevity. The estimated relationships from the benchmark are substantial; going from the empirical minimum to maximum value, both on size and diversity, cuts the predicted probability of regime breakdown in the following year by about two-thirds (for observations with mean scores on all other covariates). These relationships are robust to including or excluding data from colonies, and to controlling for country- and year-fixed effects plus time-varying covariates pertaining to income, population, natural resources income, armed conflict, degree of democracy, autocracy type (e.g., personalist vs. dominant party regimes), and state capacity. Results hold up when we measure support coalition features several years before regime breakdown or even right after the regime’s initiation, thus somewhat reducing the plausibility of our findings *mainly* being driven by regime supporters anticipating an imminent regime breakdown and jumping ship.

When assessing more nuanced patters, both the diversity and (especially) size findings hold up fairly well in different time periods and sub-samples that include only autocratic regimes. For sub-samples including democracies, coefficient sizes are typically of the same sign and size, but insignificant at conventional levels. Further tests suggest that the relationships are monotonic; regardless of initial levels, increasing coalition size and diversity consistently go together with reduced probability of breakdown. Yet, in line with work on ethnic inclusion and exclusion in sub-Saharan Africa (Roessler, 2011), we find indications that increased coalition size may limit certain types of breakdown (e.g., from popular uprisings) but possibly spur others (e.g., coups d’´etat, although these latter results are statistically insignificant). Higher coalition diversity corresponds to lower chances of coups and self-coups, in particular, and does not correspond to higher chances of any breakdown type. Although our global and historical approach cannot allow us to firmly identify causal effects, one *plausible* interpretation of our results is that heterogeneous support coalitions, with access to different types of power resources, facilitate regime survival by mitigating different regime threats.

## Literature Review

Regimes are constituted by the formal and informal rules that regulate who is selected to wield power (Djuve et al., 2020; Geddes et al., 2014). Regimes are often categorized by their “type”, with autocracies versus democracies as a key distinction. Yet, especially the universe of autocracies is diverse, with one widely used typology distinguishing between personalist, dominant-party, monarchic, and military regimes (Geddes et al., 2014). These distinctions matter for different outcomes; there is evidence, for example, that different regime types differ systematically in terms of expected regime duration (Knutsen & Nygard, 2015). More specific institutions in autocracies, notably legislatures, elections, and regime parties (Gandhi, 2008; Magaloni, 2006; Svolik, 2012; Wright, 2008; Wright & Escriba-Folch, 2012), have also been found to matter for different outcomes, including conflict, investment, and regime survival. Also scholars studying democracies have emphasized how institutional variation, especially focusing on the electoral system or form of government, influences outcomes from economic growth (Lijphart, 1999) to probability of democratic breakdown (Linz, 1990).

### Coalition Characteristics and Their Effects

Yet, regimes vary not only in their institutional make-up, but also in how their *coalitions of supporters* are composed. This is true both for democracies and autocracies. All leaders must rely on a core group of allies, or a coalition of different groups, to keep themselves in power (Bueno de Mesquita et al., 2003; Svolik, 2012) and the regime that they preside over afloat (Knutsen et al., 2025a). While many theoretical and large-N studies have (sometimes implicitly) considered the roles played by *some* groups of supporters backing either a specific leader or the wider regime, such as party-elites in party regimes and military elites in military regimes, few studies have separated this dimension from the institutional one and few studies assess variation in the full composition of support coalitions. How many *and* who these supporters are could influence the abilities of leaders and other key regime actors to pass particular policies, fight wars, and repress domestic adversaries. Such differences might, in turn, affect chances of regime survival.

These expectations can be grounded in existing theories of how regimes work. Svolik (2009), for example, theorizes how authoritarian survival is influenced by the relative power, and resulting power- struggle, between the leader and his “ruling coalition”. As theorized in Organski’s (1965) pioneering study, the identity of powerful actors supporting the regime, and the diversity of this coalition, may not only be shaped by structural transformations of society and the economy, but also influence what policies governments pursue. One prominent theoretical framework is offered by Bueno de Mesquita et al. (2003), who invoke the notions of “the selectorate” – defined as “the set of people whose endowments include the qualities or characteristics institutionally required to choose the government’s leadership” (p. 42) – and “the winning coalition”, which is the subset of the selectorate actually supporting the leadership and shaping policy and other outcomes.^4^ One implication from their core theoretical model is that smaller winning coalitions tilt public spending away from goods benefiting broader population segments, and towards narrowly targeted private goods.

Outcomes such as leader- and regime survival may also depend on coalition characteristics. Importantly, a small coalition may enhance the individual leader’s expected tenure in office, yet lead to slow growth, conflict, and other conditions that reduce the wider regime’s longevity (Bueno de Mesquita et al., 2001). Empirically, several studies have explored the relationship between coalition size and regime survival, or alternatively leader survival, but offer somewhat contrasting conclusions. While some find that smaller coalitions enhance durability (Bueno de Mesquita & Smith, 2009), others report more mixed evidence (Kennedy, 2009), or even that smaller coalitions make for *less* durable regimes (Morrow et al., 2008). One theoretically interesting possibility – also indicated by different authors cited above – is that coalition size affects different modes of regime breakdown differentially. For instance, when disaggregating regime threats and studying ethnic coalitions in Africa, Roessler (2011) finds that broad coalitions mitigate civil war risk but enhance coup risk.^5^

### Issues with Existing Studies on Coalition Characteristics and Regime Outcomes

We identify two outstanding issues in the literature linking coalition characteristics to regime outcomes: *First*, existing large-n studies of coalition size and outcomes such as leader or regime survival have used relatively crude proxies of coalition size. For instance, Bueno de Mesquita et al. (2003) use institutional indicators from Polity: military control (Regtype), competitiveness of executive recruitment (Xrcomp), openness of executive recruitment (Xropen), and competitiveness of participation (Parcomp). These measures cannot discriminate coalition size from other concepts figuring in plausible alternative theories of leader- or regime survival, for instance centering on executive constraints (see Gallagher & Hanson, 2015; Kennedy, 2009). Although Bueno de Mesquita and Smith (2022) have constructed and validated new and improved measures of (winning) coalition size by using institutional indicators from V-Dem, the issue remains that institutional features such as election monitoring board autonomy or barriers to opposition parties may *also* have independent effects on regime survival that are unrelated to coalition size. Insofar as particular institutions could, for example, enhance regime survival indirectly via broadening the coalition but simultaneously have negative direct effects on regime survival, the reliance on institutional proxies could be one reason why the literature is inconclusive on how coalition size relates to regime survival.^6^

*Second*, many empirical studies treat coalition members as relatively homogenous, or as “interchangeable”, (see, e.g., Kennedy, 2009). While this choice makes for more parsimonious and tractable theories and tests, support coalitions vary substantially on the social background – and thereby interests and power resources – of their members. Gallagher and Hanson (2015, p. 370) criticize the assumption of “interchangeable” coalitions. These authors refer to earlier studies of regime coalitions such as Roeder (1993), who identified the following key question for explaining various political and economic outcomes: “To whom are rulers accountable and what do these actors want?” (p. 26). Yet, large-n studies aspiring to investigate the identity or background of coalition actors have run into similar issues as discussed for coalition size; i.e., the lack of direct measures. Some studies of regime survival have relied on authoritarian regime classifications distinguishing between institutional configurations such as party regimes or monarchies (e.g., Geddes et al., 2014; Wahman et al., 2013). Another relevant and impressive dataset, on sources of leader support, is CHISOLS from Mattes et al. (2016). These data intend to capture “the nature of supporting coalitions” (p. 260) rather than coalition size. Yet also these data use a classification of autocracy types based mainly on institutional features (from Geddes et al., 2014) and on party composition of governments (in democracies). Geddes et al. (2018) theorize the role of “seizure groups”, the small groups that oust the incumbent and initiate a new regime, and its organized support base (p. 5). Still, they also use indirect measures, relying heavily on institutional features, such as dominant party characteristics, or leader characteristics, such as military rank. Granted, such classification schemes often carry relevant information about the background of key support groups (e.g., military officers partake in support coalitions of military regimes; Weeks, 2012), but they remain indirect ways to capture the identities of support coalitions. And, they can only proxy for the relevance of some groups, such as the military and party elites, but not others, such as business elites, industrial workers, or land owners.^7^

## Arguments Linking Two Regime Support Coalition Characteristics with Regime Survival

We define “regime support coalitions”, as “the/those groups that are supportive of the regime, and, if it/they were to retract support would substantially increase the chance that the regime would lose power” (Coppedge et al. 2022b, p. 140). This understanding centers on the coalition behind the broader regime rather than a particular leader. In many regimes, notably personalist autocracies, there is a strong correlation between leader removal and regime change, and the coalitions maintaining the current leader and regime overlap. In other regimes, notably democracies, the coalition supporting the wider democratic system may be quite different (and often broader) than that supporting the current president or prime minister (Knutsen et al., 2025a). Further, we widen the coalition concept relative to those centering on core regime elites (e.g., Geddes et al., 2018): Although these elites strongly influence policy-making, they, in turn, often rely on or represent wider societal groups. If these wider groups retract support for their elite “representatives” in close contact with the ruler, the regime’s survival chances falter. We therefore propose that also these wider societal groups can constitute “regime support groups”, as reflected in our measurement strategy. Having made these clarifications, we outline how two support coalition characteristics – *numerical size* and *diversity* – may influence regime survival.

### Coalition Size

The numerical size of a regime support coalition pertains to the number of included individuals, normalized by the country’s total population. Size does not equate diversity: Regimes can rely on one social group, such as peasants or industrial workers, but still have numerically large coalitions. Yet, size, as such, might influence regime survival. For example, members of small coalitions have strong incentives to be loyal to the regime and even a particular leader. Rulers with numerically small coalitions often rely on the distribution of private goods (e.g., Bueno de Mesquita et al., 2003), which are consumed by those particular individuals only. Especially in situations where there are many viable alternative coalition members and current members are easily replaced, members of small coalitions benefit from retaining the status quo.^8^ Regime transitions likely lead to situations where former coalition members are excluded from the new coalition, and thus private goods distribution. This prospect should incentivize members of small coalitions to remain loyal and work hard to preserve the existing regime.

However, other considerations give contrasting expectations about the size–survival relationship. Notably, increased regime support coalition size might stabilize the regime by incorporating and pleasing individuals who otherwise operate as “threats from the outside” (Svolik, 2012), for instance by instigating civil conflict (Roessler, 2011). Various forms of co-optation can take place to ensure such coalition broadening, ranging from the distribution of legislature or cabinet seats (Gandhi, 2008; Raleigh & Wigmore-Shepherd, 2020) to distributive policies targeting coalition members. And, the more citizens are invested in the regime, the fewer or weaker are its threatening opponents. Although regime elites can have incentives to restrain coalition size for other reasons, such as minimizing total rents distributed to coalition members, this argument predicts that regimes with larger support coalitions are more durable. Given these contrasting arguments, we have no clear *a priori* expectation on the sign of the aggregate relationship between coalition size and regime survival. The relationship might even be non-monotonic, as some mechanisms may dominate when increasing coalition size from very low levels and others from high levels.

One solution for uniting these expectations within a coherent framework is to distinguish between *types of threats* and *types of regime breakdown* (e.g., Bueno de Mesquita & Smith, 2017). It seems especially useful to distinguish between potential threats to the regime emerging from existing coalition members wanting to change the regime, and those originating in actors outside the coalition (Bueno De Mesquita, 2010; Bueno de Mesquita and Smith, 2009, 2023; Roessler, 2011; Svolik, 2012). The former actors may challenge the regime through coups d’´etat and the latter through mass uprisings. Arguments about how small coalitions should stabilize regimes through promoting loyalty are mainly relevant for explaining why coalition “insiders” do not threaten regime stability through coups. Mechanisms concerned with the inclusion or co-optation of additional citizens (for securing regime stability) mainly apply to regime breakdown through mass uprisings. After assessing the overall size–survival relationship, we will test these more nuanced expectations.

### Coalition Diversity

The *social composition* of regime support coalitions might also influence regime durability, even when holding numerical size constant, for instance because social groups differ in the amount and types of power resources they can grant the regime access to. Consider a hypothetical country where the population consists of 80% peasants, 10% industrial workers, 4% land-owners, 4% military officers and soldiers, and 2% business elites. For this country, three potential regime support coalitions consisting of, alternatively, 1/8 of peasants *or* all industrial workers *or* all land-owners, military members, and business elites combined comprise 10% of the population. Yet, these three coalitions may yield different rates of regime survival, reflecting the different power resources of the groups partaking in the coalitions. Particular social groups may, generally, be more important for regime survival than others. The military, for example, could be particularly important for enhancing repressive capacity and mitigating external threats. Despite the likely relevance for regime survival, we do *not* focus on the presence or absence of one *particular* social group in this paper, other than controlling for the coalition participation of 14 specific groups in empirical tests when assessing our main hypotheses. Instead, we here consider the more general notion of coalition *diversity*, and focus our discussions and subsequent empirical tests somewhat more specifically on *the number of social groups* that partakes in the regime support coalition.^9^

We anticipate that diverse coalitions are conducive to regime survival. This expectation builds on the assumption that regime durability is shaped by the regime’s access to a wide range of power resources, which may be used to mitigate different threats. And, following insights developed by, e.g., Korpi (1983, 2006) and Slater (2010), we further assume that different social groups command different types of power resources. Business elites command, inter alia, financial resources, peasants command manpower, state bureaucratic elites command organizational capabilities, party elites offer access to organizations with linkages to key brokers and civil society organizations, and so forth. Different resources are more effective in mitigating different threats and for governing in different contexts. During economic crises, easy access to credit is critical to continue spending on various programs aimed at co-opting potential contenders and mitigating grievances. During armed uprisings or foreign interventions, having firm control over military resources could help a regime to fend off such threats. Commanding support from particular local leaders or ethnic and religious groups could help bolster legitimacy and stem threats in particular geographic regions. Other plausible examples can easily be added.

Including members from several social groups in the support coalition might thus come with large benefits, both for relatively democratic and relatively autocratic regimes. Indeed, we anticipate that increasing the number of social groups represented enhances survival even when holding coalition size constant, since we anticipate *decreasing returns to scale* in how effective most power resources are for mitigating regime threats. Improved access to financial resources, for example, may always reduce probability of regime breakdown, but the marginal effect is presumably larger where such resources are initially scarce and regimes can barely afford to repress or co-opt the most dangerous threats. In overtly simplified terms, having access to a moderate amount of power resources of different kinds is often preferable to having a lot of one power resource – access to diverse power resources provides regimes with more flexibility in identifying the most effective combination of measures to counter different types of threats (which often have very different vulnerabilities). Hence, regimes that have more diverse bundles of power resources should be more stable than regimes displaying more extreme allocations. If so, regimes that draw on the support from different social groups should be better at mitigating various threats than regimes with equally sized, but more homogeneous, coalitions.

The insight that diverse coalitions enhance regime survival, inter alia through granting regimes access to diverse power resources which allow them to mitigate different types of threats, is supported by in-depth studies of regimes in different countries. Notably, Slater (2010) proposes that in Southeast Asia, authoritarian regimes undergirded by more diverse (elite) coalitions mitigate (mass) threats since they command power resources of different kinds. Slater divides these power resources into three groups, namely “coercive”, “remunerative” and “symbolic”, proposing that “the more of these power resources regimes lack, or lose over time, the more vulnerable they become to anti-regime mobilization and democratic transitions” (p. 16). Further, as highlighted by Raleigh and Wigmore-Shepherd (2020) in the African context, “big tent” coalitions that co-opt (rivaling) elite groups, e.g. through cabinet posts, mitigate different threats to regime stability, also because these elites can command support and resources from different parts of the population.

Beyond ensuring a broad basket of power resources to fend off external threats, broadening the support coalition to cover different groups, for instance through co-optation, may also more directly “remove” threats by turning potential enemies to supporters. Such inclusion, at least, reduces the chances that these groups’ power resources are used to challenge the regime. This more direct benefit of adding groups to the coalition may also be relevant for the inclusion of non-elite groups. For example, industrial workers presumably constitute a particularly acute threat to autocratic regimes, due to their potential to disrupt economic activity (Kim & Gandhi, 2010). Incorporating industrial workers could thus mitigate one key threat by removing these workers from the ranks of the opposition. Also for democracies, incorporating additional groups in the regime’s support coalition expectedly bolsters regime survival in similar ways. Democracies vary considerably in the extent to which elite groups are part of their support coalitions (Albertus & Menaldo, 2018). Coups d’´etat – often conducted by military officers and bank-rolled by disgruntled economic elites – make up one prominent threat to democracies (Svolik, 2015), and in-depth case studies propose that achieving comprehensive elite coalitions behind a democratic regime’s institutions and norms is vital for mitigating coup risk and consolidating democracies (Higley & Gunther, 1992). Despite making up relatively small population shares, elite groups such as landowners, business elites, and military officers – when not counted among the regime’s support groups – can pose severe threats to democracies, insofar as they can potentially use their ample power resources to challenge the regime.

Nonetheless, there are caveats and counter-arguments to the argument above and the direction of the overall relationship between coalition diversity and regime survival thus remains an open empirical question. Regime survival is enhanced by strong coalition loyalty, which in turn relies on distribution of economic benefits to supporters. If regimes distribute targeted private goods for keeping power, it may be costly and hard to maintain this strategy if the coalition consists of groups with very different social concerns and demands. Another counter-argument pertains to the importance of maintaining trust and friction-free cooperation within the coalition to ensure continued regime support. If social group heterogeneity reduces trust and effective cooperation (e.g., Alesina & La Ferrara, 2005), broader coalitions could induce within-coalition conflict and gridlock, setting the regime at risk. Further, broad coalitions could destabilize regimes by empowering groups – e.g., through expanded networks and access to resources – that may back the regime at present, but which may ideally prefer to replace it in the future via a coup (Roessler, 2011).

## Measures and Model Specification

### Dependent Variable: Regime Breakdown

To identify regime units and when regimes break down, we use in-house coded data from the Historical Regimes Data (HRD) from Djuve et al. (2020), which covers more than 2000 distinct regimes from about 200 countries. HRD codes all regimes that existed from the first year the country enters the V-Dem dataset – for France, for example, this is the pre-revolution Ancient Regime which held power on January 1st 1789 – to 2021. Regimes are defined as the set of formal and informal rules that are essential for selecting political leaders, and for maintaining them in power. A regime breakdown is thus defined as a substantive *change* to these rules. The broad definition implies that HRD captures not only transitions between autocratic and democratic regimes, but also between different regimes that are about equally (un-)democratic. Episodes of substantial shifts in the rules for selecting leaders are thus considered regime breakdowns when, say, a dominant-party autocracy replaces an absolutist monarchy but also when the new regime is of a similar “type” as the old one, such as one military regime replacing another via a coup.

Specifically, we use the HRD measures on regime (start- and) end-dates to construct our core dependent variable of regime breakdown and covariates on regime duration. In our benchmark sample, the median and mean observations on regime duration are, respectively, 11.7 and 22.2 years, and about 6.9% of country-years experience at least one regime breakdown. For our extended analysis, we also rely on a 14-category scheme of the primary process leading to regime breakdown, enabling us to distinguish breakdowns occurring from, e.g., coups d’´etat, popular uprisings, and international intervention (see Djuve et al., 2020 for details).

### Independent Variables: Regime Support Coalition Size and Diversity

The regime support coalition size and diversity indicators were designed by the authors and collected with the help of more than 1000 country-experts and using the V-Dem infrastructure (Coppedge et al. 2022a). While adequately responding to such questions requires expert knowledge, experts should also have similar underlying concepts in mind when coding. Ensuring perfect consistency is impossible, but to enhance it, all V-Dem experts were presented with clarifications of the relevant concepts (“regime”, “support coalition”, etc.) plus dates and identity of the country’s political regime (from the in-house- coded HRD data). We refer to the V-Dem codebook’s Regime survey section for details, but highlight that experts could code support coalition features as time-varying also *within* regimes.

We are under no illusions that these new expert-coded measures are flawless, despite our best efforts to enhance validity and comparability across time and space. For instance, Knutsen et al. (2025a) report and discuss several reliability and validity tests of these measures, showing, for example, that observations coded by fewer country experts are less reliably measured than those coded by more experts and that uncertainty in the regime support coalition size measure is higher for intermediately sized coalitions. One might also worry about different types of biases in the expert coding for these measures, especially since a group’s “influence on regime survival” is complicated to evaluate and impossible to observe directly. Thus, even historically very knowledgeable experts may use different cues and heuristics when coding the size or number of groups included in the support coalition, and these cues may be related to how and when the regime broke down. A regime that ended via a large popular revolt in year *t* + 5 might, for instance, *be coded* as having smaller and less diverse support coalition in year *t* than a regime with an identical regime support coalition, but which happened to survive until *t* + 30. In this case, coding bias might generate a spurious correlation between regime size and diversity, on the one hand, and regime duration, on the other. Yet, these kinds of heuristic-coding biases must be shared by several experts of a country to considerably influence results (in which case, the bias might also increase inter-coder reliability metrics). We cannot rule out this possibility, especially since country experts may often base their coding on the same prominent historical events and key sources. Yet, systematic tests on related post-hoc coding biases for other V-Dem variables, assessing how notable current events influence coding of past years, have shown systematic, though substantially small, biases for some variables (Weidmann, 2024). With this caveat in mind, we note that the support coalition measures turn out to perform fairly well across a range of convergent validity- and other tests, and aggregated country-year scores are not particularly sensitive to, e.g., randomly leaving out one coder for each observation (Knutsen et al., 2025a).

Equally important, we believe that these are informative measures of concepts that are of great theoretical interest, and they differ from, and in important ways improve upon, existing cross-national measures of similar concepts. While previous contributions have used different proxies to model features of coalitions (variously defined), such as measures of institutions or sectoral composition of the economy, our measures tap more directly into the size and social composition of support coalitions. The measures were originally coded for all observations in Historical V-Dem, approximately 85 countries with the modal time series being 1789–1920 (see Knutsen et al., 2019), and has later been expanded to cover the entire V-Dem time series. In V-Dem v.12, they are coded for almost 26,000 country-year observations across 194 polities. The longest time series extend from 1789–2020 and the modal time series from 1900–2020. The mean number of country experts coding each observation is around 5.

Regarding coalition size, the relevant V-Dem question asks: “In total, how large is the percentage share of the domestic adult (18+) population that belongs to the political regime’s supporting groups?”. The accompanying clarification states that experts should “take into account the total size of the/those groups that are supportive of the regime, and, if it/they were to retract support would substantially increase the chance that the regime would lose power” (see Appendix A.1 for full clarification). The answer categories range from “0: Extremely small (About 1% of the population or less; examples of this could include regimes supported by – and needing the support from – a handful of higher-rank military officers, or by only a royal council and a few hundred landowners)” to “4: Large (More than 30%; examples of this could include regimes supported by – and needing the support from – large ethnic groups (and then not only the elites/leaders of such groups), or by rural working classes in rural societies.)”. Once this five-point ordinal measure has been coded by country-experts, all scores are processed through V-Dem’s measurement model (Pemstein et al., 2015), which adjusts scores for differences in scale perception and gives more weight to the coding of more reliable experts. This measurement model draws on various types of information, including anchoring vignettes, cross- country coding, and coding for overlapping years between contemporary and historical experts, to make expert scores comparable across space and time. The model generates a latent variable measure, which ranges from −3.90 (Burkina Faso under French colonial rule from 1919-31) to 2.93 (Greece right after democratization in 1975-6), with a median, mean, and standard deviation of, respectively, −0.03, −0.08, and 1.52.

**Figure 1.**
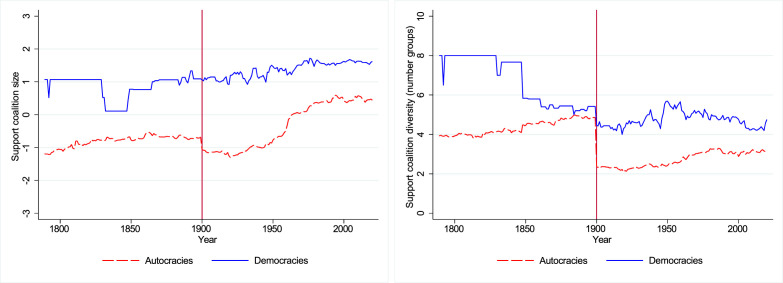
Average regime support coalition size (left) and diversity (right), by regime type, 1789–2020.

The left plot of Figure 1 displays the historical development of average support coalition size, globally, by regime type. The regime categorization registers countries as democratic if they score 4 or higher on the Lexical Index of Electoral Democracy (LIED; v.6.3) by Skaaning et al. (2015). Hence, democracies are here operationalized as regimes with contested multi-party elections, whereas autocracies are regimes without multi-party elections or where such elections fail minimal standards of fairness. First, we note that democracies, throughout modern history, have had far larger support coalitions than autocracies, on average (yet, there is ample within-regime variation, especially among autocracies; see Knutsen et al., 2025a). Second, we note a strong, positive trend in the global average for autocracies since the mid-20th century, whereas the average democratic support coalition has grown more modestly. Third, we note a downward shift in 1900, also demarcated with a vertical, dotted line, for the autocratic regimes. This shift is due to the V-Dem sample expansion in 1900; almost all 50 polities that enter in 1900 were African and Asian colonies, governed as autocracies where coalition size was typically low. The changing sample makes it extra important to control for both country- and year-fixed effects in our regression analysis, and we also run our analyses on pre- and post-1900 samples to assess robustness.

To construct our measure of coalition diversity, we use the identity of regime support group measures, capturing the social groups partaking in the support coalition. V-Dem experts – responding to: “[w]hich groups does the current political regime rely on in order to maintain power?”– were presented with a 14-category scheme of potentially relevant groups. They were further instructed that, to be counted, groups should be “supportive of the regime, and, if it/they were to retract support would substantially increase the chance that the regime would lose power”. The 14 relevant groups are *the aristocracy*, *agrarian elites*, *party elites*, *business elites*, *the state bureaucracy*, *the military*, *ethnic or racial group(s)*, *religious group(s)*, *local elites/chiefs*, *urban working classes*, *urban middle classes*, *rural working classes*, *rural middle classes*, and *a foreign government or colonial power*. A multiple selection variable allows experts to include *any relevant support group*.^10^

Since several experts code one observation, we need aggregation rules for arriving at final scores. For each group, experts code 1 if the group is considered part of the coalition and zero otherwise. In V-Dem, expert scores are aggregated by the mean. By illustration, if three of five experts consider party elites to be in the coalition, the corresponding V-Dem variable is scored (3 ∗ 1)*/*5 = 0.6. Building on these variables, we construct dummies scored 1 if the V-Dem score is ≥0.5. Thus, we may think of a 1-score on our dummies as signifying a group’s presence in the regime support coalition – which applies if at least half of the experts considered the group present – and zero signifying its absence. Next, we use these dummies on coalition participation to build our main diversity measure. Specifically, support coalition diversity is a count measure summing scores from all 14 dummies to capture the number of groups backing the regime.

By requiring that at least a majority of experts agree that a group is part of the support coalition, we set a moderate threshold for registering support groups. A “regime support group” is an inherently hard concept to measure precisely, and inter-coder agreement is unlikely to be high.^11^ Despite our definitions and clarifications, experts presumably operate with different thresholds for considering when a group displays the sufficient level of support or the sufficient influence to register as a relevant regime support group. The majority-of-experts rule makes us less sensitive to the judgement of single experts compared to a rule requiring expert consensus. In the latter case, experts with very high thresholds for coding support groups (or simply coding erroneously) would have “veto power” over the country-level scores. Thus, our benchmark measure, presumably, also enables us to pick up groups with considerable influence over the regime, without requiring that influence must be extremely high. The results presented below for the diversity measure are, however, robust to different operationalization choices, such as altering the threshold for when a support group is scored as present. More specifically, results are very similar when we require that 40%, 45%, 55% or 60% – instead of the benchmark 50% of expert coders – code a group as being a support group (see Appendix Table A4).

Figure 1 shows that the average number of groups increased from around 4 in 1789 to around 4.5 in 1899 for autocracies. For democracies the average number dropped around the “year of revolution”, 1848, but this is mainly due to the expansion in number of democracies (only USA is counted as democratic in 1800 whereas six countries are counted in 1850). Once again, the inclusion of African and Asian colonies in 1900 lowers the global average for autocracies, from around 5 to around 2.5, reflecting the very limited diversity of colonial regime coalitions. We note here that “regimes” and “support coalitions” for these cases refer to the administration and groups located in the specific colony, and not in the metropole, which is not only territorially distinct, but also governed differently. After the sample-expansion in 1900, average coalition diversity for autocracies has experienced a limited, gradual increase until the present. Yet, these global averages mask substantial cross-country variation (for additional descriptive statistics on cross-country-, cross-regime-, and within-regime variation on this measure, see Knutsen et al., 2025a). In 1800, for example, Tripoli (Libya) and Nejd (Saudi Arabia) had only one support group each in their coalitions, whereas the United States and Persia (Iran) had eight. In 1900, the empirical range extended from 1 group (mostly colonies) to 8 (New Zealand and Austria). In 2000, Suriname and Belgium registered 10 groups, whereas 19 countries had only one. There is only a *moderate* correlation between diversity and size (.08, .50, and .41 for 1800, 1900, and 2000, respectively, and .33 in the full sample), reflecting that these are distinct concepts.

### Benchmark Specification

Our dependent variable is a regime breakdown dummy, coded 1 if a regime, as defined by HRD, breaks down during the relevant calendar year, and zero otherwise (for details, see Djuve et al., 2020). Following several previous studies on regime breakdown, our benchmark specification is a logit model that incorporates duration dependence, where we include linear, squared and cubed polynomials of time since regime birth (following Carter & Signorino, 2010). A substantive motivation for including these polynomials is that regime fragility is a non-linear function of how long the regime has endured (Svolik, 2012), and coalition size and diversity could also change systematically across a regime’s life- span. We use country-year as unit of analysis and whenever there are multiple regimes per year we register the information pertaining to the last one; we forward-lag the dependent variable by one year in the benchmark, and thus make sure that we are (predominantly) capturing the same regime with our covariates and outcome measure, also during the regime’s first year.^12^ In additional tests reported in Appendix A.2, we try out even longer time lags and we also report specifications using the size and diversity scores right after the regime’s inception; our main results are robust to these changes. Results are also robust to using Linear Probability Models (LPM) instead of Logit and to using measures of regime duration and breakdown from Geddes et al. (2014) instead of HRD. We always cluster errors by country to handle panel-specific autocorrelation.

We include regime support coalition size and diversity jointly in the benchmark to disentangle their relevance for regime breakdown. But we also test specifications where they enter individually. We consciously opt for a sparse set of benchmark controls since the actors included in the support coalition may not only be affected by, but also influence, factors such as short-term growth or presence of armed conflict. If so, controlling for such features will “hold constant” important mediating variables, and thus block off part of the relevant, total effects of support coalition characteristics on regime survival. Granted, growth and conflict may also be confounders, as they could also causally affect coalition features; therefore, we introduce such controls in robustness tests. In particular, we find it hard to conclude on whether democracy is an appropriate control or not, and we therefore estimate several specifications both with and without controlling for democracy levels.

We always include country-fixed effects to pick up stable, country-specific confounders pertaining, for example, to resource endowments, geographic characteristics or persistent societal norms. This is made feasible by our long time series, which ensure multiple regime breakdowns per time series (on average 8.0 per time series in the benchmark sample) and substantial over-time variation in support coalitions characteristics; the between- and within-panel standard deviations are, respectively, 0.9 and 1.1 for coalition size and 1.7 and 1.6 for coalition diversity. We also include year-fixed effects to model common (and likely non-linear; see Djuve et al., 2020) global time trends and short-term shocks to probability of breakdown. Our two final benchmark controls reflect that demographics and economic development may shape the number and types of actors in the support coalition (e.g., Organski, 1965) *and* regime survival (e.g., Djuve et al., 2020). We control for Ln GDP per capita, using extensive-coverage data from Fariss et al. (2022). These authors provide GDP estimates constructed by a dynamic latent trait model run on data from several historical and contemporary sources. We also control for Ln population, culled from V-Dem and extrapolated by assuming constant annual population growth rates to minimize listwise deletion. By using these data, we extend the time series of investigation from 1789–2020 and include about 19,500 regime-year observations from around 170 countries.

Despite the control strategy laid out above, and the different robustness tests presented in the next section trying to deal with various sources of confounding and endogeneity, we cannot account for all such sources. Given the nature of the questions we investigate and the data and design we rely on, strict causal identification is difficult to achieve; our results should thus be interpreted as partial correlations that are robust to accounting for several sources of confounding. Our theoretical arguments pointing to causal effects of, respectively, coalition size and diversity on regime duration describe very plausible candidate mechanisms that may generate these observed correlations, but we cannot exclude that other factors are driving, or at least contributing to, them.

## Results

**Figure 2.**
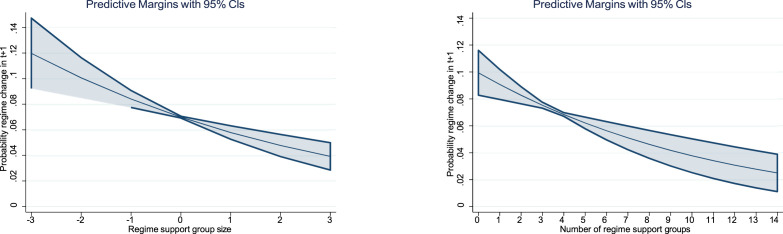
All regimes: Predicted probabilities of regime breakdown, for different sizes of regime support coalitions (left panel) and levels of coalition diversity. Predictions from benchmark model (Model 3, Table 1), for observations with mean scores on all covariates.

Models 1.1 and 1.2 in Table 1 are reduced versions of our benchmark where we only include one of the coalition size (1.1.) or diversity (1.2) measures, whereas Model 1.3 is the benchmark including both measures. Regardless, the size and diversity coefficients are both negative and statistically significant at the 0.001 level. The findings from Model 1.3 are especially notable; larger coalitions systematically correspond to lower predicted probability of breakdown, even when holding the number of participating support groups in the coalition constant, and similarly for more diverse coalitions when holding coalition size constant. The estimated coefficients indicate substantially sizeable relationships. This is illustrated by Figure 2. When going from −2 (approximately 10th percentile observation) to +2 (approximately 90th percentile) on the coalition size measure, with other covariates in Model 1.3 at their means, the predicted probability of regime breakdown in the following year drops from 0.10 to 0.05. Likewise, when going from one to seven groups in the coalition, the predicted probability shrinks from 0.09 to 0.05.

**Table 1.** Features of Regime Support Coalitions and Regime Breakdown.

Model	1.1	1.2	1.3	1.4
Regime support coalition size	−0.266***		−0.214***	−0.178***
	(−5.90)		(−4.35)	(−3.34)
Regime support coalition diversity		−0.141***	−0.110***	−0.0905**
		(−5.32)	(−3.84)	(−3.02)
Ln GDP p.c.	−0.454***	−0.521***	−0.475***	−0.451***
	(−4.15)	(−4.80)	(−4.33)	(−3.84)
Ln population	0.136	0.153	0.173	0.188
	(1.14)	(1.34)	(1.46)	(1.62)
Electoral democracy index				−0.718*
				(−2.12)
Cubic regime duration controls	Y	Y	Y	Y
Country dummies	Y	Y	Y	Y
Year dummies	Y	Y	Y	Y
*R*^2^ (pseudo)	0.116	0.115	0.119	0.119
ll	−4428.4	−4412.5	−4358.5	−4271.0
N	19,502	19,417	19,256	18,855

Notes: + *p <* .10; *p < .05; **p < .01; ***p < .001. Coefficients from logistic regressions with t-values in parentheses. Standard errors are clustered on country and unit of analysis is country-year. Dependent variable is dummy for regime breakdown, measured in *t* + 1 (all covariates measured in *t*).

Model 1.4, Table 1 adds V-Dem’s Electoral democracy index to the benchmark. This is an important test, as democracy is not only associated with larger and more heterogeneous support coalitions (as shown above) – democracies are also particularly stable regimes. Both coalition size and diversity remain negative and significant at least at 1% when controlling for democracy, even though both coefficients and their t-values are somewhat attenuated. We also ran numerous other robustness tests. Most of them are reported in Appendix A.2, but a selection of important ones are presented in Table 2.

**Table 2.** Selected Robustness Tests: Top Column Specifies Variation on Benchmark (Model 3, Table 1).

Type test	OLS	No country-FE	No year-FE	Add controls	Add group id.	3-yr lag	10-yr lag	Reg.’s 1st yr
Model	2.1	2.2	2.3	2.4	2.5	2.6	2.7	2.8
Reg. sup. coal. size	−0.0166***	−0.184***	−0.234***	−0.196*	−0.180**	−0.263***	−0.162**	−0.183***
	(−4.82)	(−5.64)	(−5.14)	(−2.19)	(−3.15)	(−4.69)	(−2.64)	(−3.69)
Nr. reg. sup. groups	−0.00577***	−0.0626**	−0.129***	−0.228***	0.131	−0.0768*	−0.0513+	−0.105***
	(−3.36)	(−2.99)	(−4.62)	(−4.15)	(0.90)	(−2.57)	(−1.72)	(−3.35)
Ln GDP p.c.	−0.0161**	−0.190***	−0.398***	−0.481+	−0.389***	−0.416***	−0.432***	−0.468***
	(−2.92)	(−3.99)	(−4.56)	(−1.66)	(−3.71)	(−3.90)	(−3.50)	(−4.08)
Ln population	0.000985	0.00135	0.245**	0.571+	0.147	0.132	0.0860	0.195
	(0.16)	(0.06)	(3.23)	(1.80)	(1.13)	(1.05)	(0.63)	(1.62)
GDP p.c. growth				−0.0638***				
				(−4.05)				
Ln nat. res. inc. p.c.				−0.0253				
				(−0.32)				
Urbanization				−0.0465				
				(−0.05)				
Internat. conflict				0.131				
				(0.69)				
Internal conflict				−0.0163				
				(−0.10)				
Impartial admin.				−0.0876				
				(−0.97)				
Dom. party regime				−0.349				
				(−1.13)				
Personalist regime				0.385+				
				(1.84)				
Military regime				0.0551				
				(0.24)				
Autocratic monarchy				0.356				
				(1.14)				
Business elites					−0.412*			
					(−2.27)			
State bureaucracy					−0.411*			
					(−2.44)			
Urban middle classes					−0.480*			
					(−2.04)			
Rural working classes					−0.558*			
					(−1.97)			
Cubic regime duration	Y	Y	Y	Y	Y	Y	Y	Y
Country dummies	Y		Y	Y	Y	Y	Y	Y
Year dummies	Y	Y		Y	Y	Y	Y	Y
Dummies all sup. Gr.					Y			
*R*^2^ (pseudo)	0.247	0.0922	0.0775	0.137	0.123	0.189	0.190	0.122
ll	−236.6	−4526.5	−4572.9	−1815.1	−4338.3	−4278.8	−4031.6	−3858.6
N	19,954	19,805	19,403	7,506	19,256	19,529	18,385	16,629

Notes: + *p <* .10; *p < .05; **p < .01; ***p < .001. T-values in parentheses. Standard errors clustered on country and unit is country-year. Dummies for all 14 support groups enter Model 4, but coefficients for dummies with p *>* .05 are omitted from table.

### Robustness Tests

Model 2.1 in Table 2 – an OLS version of the benchmark – exemplifies that results are robust to choice of estimator (see also Appendix Table A3). This particular linear probability model predicts that a one unit increase on the support coalition size measure reduces probability of regime breakdown by about 0.017, approximately the reduction in breakdown probability predicted from adding three new groups to the support coalition. Further, Models 2.2 and 2.3 alter the benchmark logit model (Model 1.3, Table 1) by omitting, respectively, the country- and year dummies. Both coalition size and diversity are robust, and so are they to dropping both sets of fixed effects simultaneously as well as the other covariates (Appendix Table A5). This assuages concerns that our results are artifacts of colinearity or post-treatment bias from including the benchmark controls.

Results are also robust to adding other potentially relevant controls. More specifically, both coalition size and diversity remain significant at least at 5% in Model 2.4, which expands the benchmark by adding GDP p. c. growth in year t, (Ln) natural resources income p. c., urbanization rate, ongoing internal armed conflict, ongoing international armed conflict, impartial and rule-following administration, and autocratic regime type dummies.^13^ Appendix Table A6 shows that our main independent variables are robust also when adding these extra covariates individually. These tests are important as the benchmark results could have been be due to characteristics such as natural resource wealth or high growth signaling to actors considering to join the support coalition that the regime is strong and expected to endure, and therefore worth supporting. Furthermore, by controlling for regime type dummies, we address the possibility that regimes with different institutions differ both in coalition characteristics (see Knutsen et al., 2025a) and durability (Geddes et al., 2018). For example, so-called dominant party autocracies may both be longer-lived and have more extensive and heterogeneous coalitions than military regimes.

Further, we noted how the determinants of regime breakdown may differ between independent and non-independent states. Including observations from colonies and semi-independent entities in the estimation might influence estimates and errors, for instance because metropole-level factors may largely determine colony-level regime duration. However, results are robust to excluding all colonies and re- estimating specifications for independent states only (see Table A2). One remaining concern is that the coalition size and diversity findings are due to specific groups participating in large or diverse coalitions. While general notions such as coalition size and heterogeneity may influence regime survival, so could more particular features, such as whether the included groups represent urban/industrial or rural/a- grarian interests (e.g., Ansell & Samuels, 2014) or certain economic, military, or administrative elites. Indeed, the diversity coefficient turns insignificant in Model 2.5 that adds all 14 support group dummies to the benchmark. However, this is a very demanding specification, which is associated with colinearity and post-treatment concerns.^14^ Importantly, the diversity finding is robust (at 1%) in *all* specifications where only one of these dummies enter at the time (Appendix Table A7). The coalition size measure is robust both in Model 2.5 and in these latter, more parsimonious specifications.

**Figure 3.**
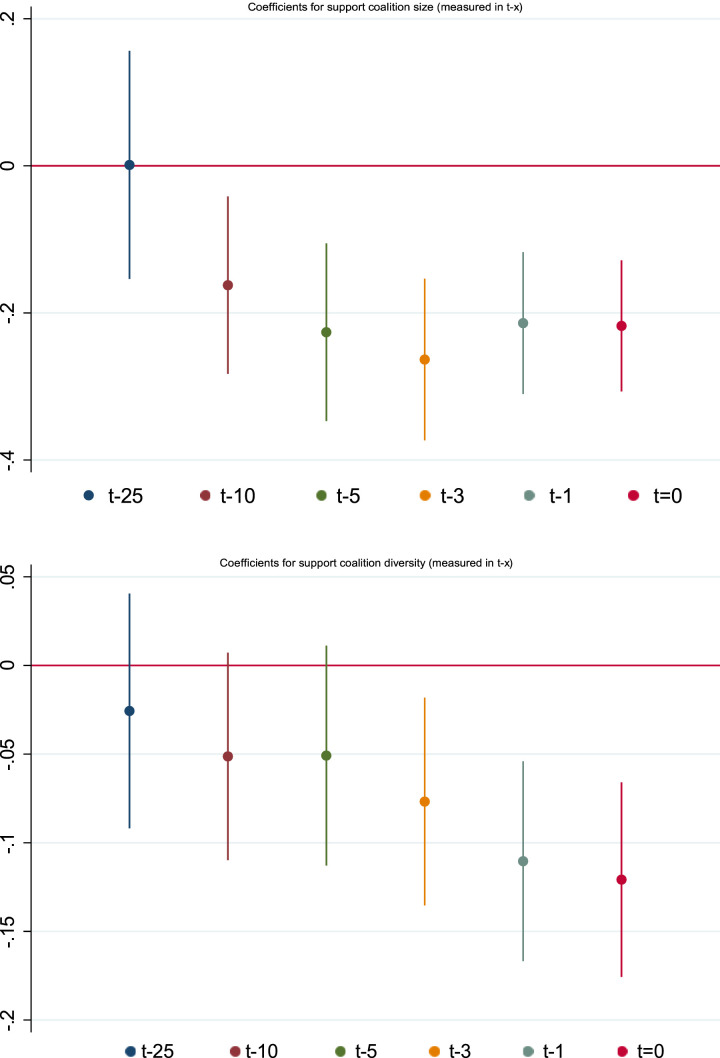
Results from versions of benchmark (Model 3, Table 1) where covariates are measured *t* − *x* years before regime breakdown. Coefficient estimates (with 95% CI) are shown for support coalition size (top) and support coalition diversity (bottom).

Finally, we increase the lag-length between when regime breakdown and our covariates are measured. The benchmark’s 1-year could make us vulnerable to mainly capturing snowballing effects (that may or may not be of causal relevance to breakdown), as regime supporters could possibly “leave the sinking ship” if they believe a regime collapse is near. Coups and revolutions are inevitably hard to predict (e.g., Kuran, 1989), but regime insiders presumably sometimes have access to relevant information on regime fragility and changes in threat-levels. While increasing the time-lag attenuates the size and diversity coefficients, they still systematically predict regime breakdown when measured, for example, three (Model 2.6) and even ten (Model 2.7) years before the outcome (see also Figure 3). Indeed, results hold up even when we measure coalition characteristics only based on scores from the first year after the regime’s inception (Model 2.8). These results do not ensure that our benchmark results reflect causal effects running from coalition characteristics to regime breakdown, but they do make such an interpretation somewhat more plausible.

## Extensions: Heterogeneity Across Time and Regime Type, Non- Monotonic Relationships, and Specific Modes of Regime Break- down

Despite the robustness of the coalition diversity and (especially) size findings in specifications reported so far, they all assume that relationships are homogeneous and monotonic. Yet, we are using global data from different centuries and from different regime types, which makes it likely that the results mask heterogeneity. Further, our theoretical discussions highlighted candidate mechanisms that should pull in the opposite direction of our aggregated results. Large-coalition regimes could, for example, be more vulnerable to regime-ending coups, despite being less vulnerable to other sources of regime death such as popular uprisings. Multiple mechanisms pointing in different directions could also lead to non-monotonic relationships; increasing coalition size could, for example, increase breakdown probability if the initial coalition is very small and reduce it if the initial coalition is larger. We explore such possibilities in this section. Before we discuss the results, let us caution that we run quite demanding specifications, and limiting the sample will likely increase standard errors. Similarly, specifications allowing for non- monotonic relationships are more sensitive, and could lead to excessive curve-fitting. Hence, results from this section should be interpreted with particular caution.

Appendix Table A8 reports results for specifications run on, respectively, the Historical V-Dem part of the time series, 1789–1899, and 1900–2020. Coefficients are consistently negative in both sub- samples, although point estimates and t-values are typically much higher for both diversity and size in the post-1900 samples. Still, given the much more limited number of pre-1900 observations, the sizeable point estimates and decently sized t-values (at least for coalition size) indicates that we cannot strongly conclude that the relationships were absent in the early parts of modern history. We also replicate the findings by using regime duration and breakdown data from Geddes et al. (2014), which extend from 1946–2010, in Appendix Table A9. Our main results are robust also to using these measures (or to using our main HRD measures in the 1946–2010).

Next, we assess whether results differ across autocracies and democracies, once again using a 4-score on Skaaning et al.’s (2015) LIED as cut-off. In Table 3, we re-run our benchmark on autocratic (Model 4.1) and democratic (4.2) observations, and Appendix Table A10 shows results for other specifications from Table 1. Results are very robust for the autocratic sub-sample (see also Table A11), indicating that larger and more diverse coalitions prolong the life of autocratic regimes. The differences in predicted probabilities of regime breakdown are also substantial and quite similar for the autocratic and full samples (c.f. Figure 2 and Appendix Figure A.1).

**Table 3.** Replicating the Benchmark (Model 1.3, Table 1), by Regime Type.

Sample	Autocracies	Democracies
Model	4.1	4.2
Regime support coalition size	−0.223***	−0.314
	(−3.70)	(−1.36)
Regime support coalition diversity	−0.0969**	−0.0676
	(−2.82)	(−0.80)
Ln GDP p.c.	−0.258*	−0.435
	(−2.05)	(−1.00)
Ln population	0.0404	1.210*
	(0.29)	(2.36)
Cubic regime duration controls	Y	Y
Country dummies	Y	Y
Year dummies	Y	Y
*R*^2^ (pseudo)	0.112	0.231
ll	−3451.9	−617.4
N	13,015	3845

Notes: + *p <* .10; *p < .05; **p < .01; ***p < .001. Coefficients from logistic regressions with t-values in parentheses. Standard errors clustered on country and unit is country-year. Dependent variable is dummy for regime breakdown, measured in *t* + 1 (all covariates measured in *t*).

Results are not as clear for democracies, as the logit coefficients turn out insignificant (although OLS versions produce significant results for coalition size, even in democracies, see Table A3). However, coefficients are consistently negative and often larger in size in the sub-sample of democracies. Yet, given the large standard errors, we cannot conclude that coalition size and diversity is systematically related to democratic regime breakdown. The larger uncertainty for democracies could reflect several factors, for instance the fewer observations or larger measurement errors for our concepts of interest in this context. Concerning the latter point, we anticipate that some country-experts have a harder time conceptualizing what the relevant regime units and support coalitions are in democracies (hence our added clarifications in the survey on how to interpret these concepts in democracies). However, we cannot reject the hypothesis that the democracy results are similar to the autocracy- or full-sample results either, and we therefore proceed with the full sample comprising both autocracies and democracies.

**Figure 4.**
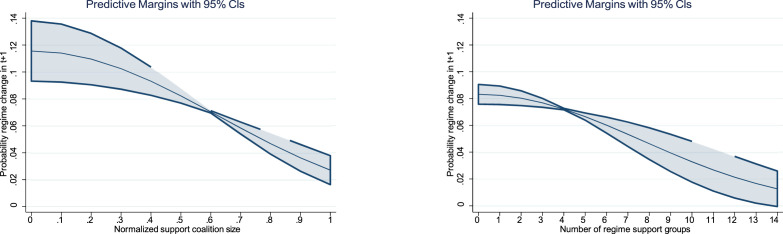
Assessing non-monotonic relationships: Predicted probabilities of regime breakdown, across size (left panel) or heterogeneity (right panel) of regime support coalitions. Predictions are from a version of Model 3, Table 1 including squared terms on support coalition size and support coalition diversity. All covariates at their means.

Next, we allow for the relationships to be non-monotonic by adding squared terms of coalition size (normalized to 0–1) and diversity to our benchmark, Model 1.3. Figure 4 plots the results for observations with mean values on the covariates. Figures presenting results from a similarly extended Model 1.4, Table 1, which controls also for squared democracy levels, as well as OLS specifications are included in Appendix A.2. We find no evidence of non-monotonicity across these specifications; regime breakdown probability is consistently decreasing in both coalition size and diversity.

**Figure 5.**
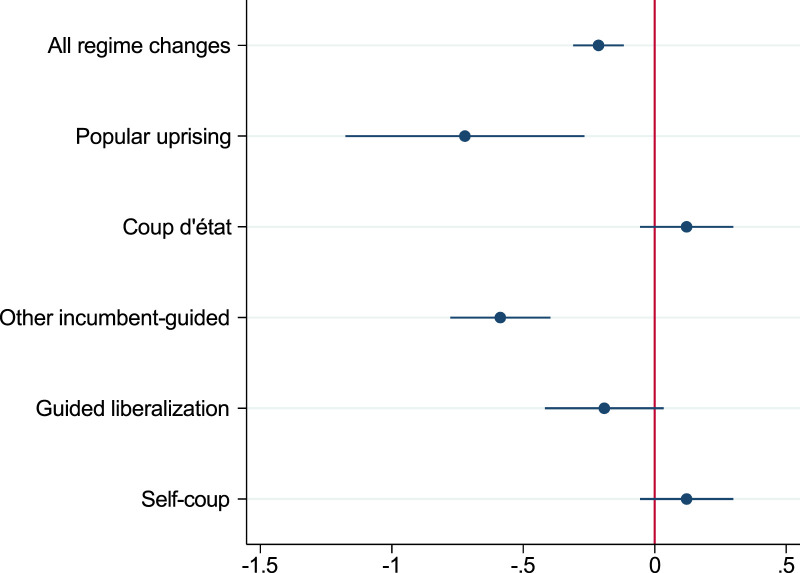
Coefficient estimates for regime support coalition size. DVs: Types of regime breakdown. Model specifications based on Model 3, Table 1.

To assess whether larger coalitions enhance some regime threats but mitigate others, we use the “regime breakdown mode” coding in HRD to distinguish between different processes leading up to the breakdown event. In particular, we discussed how larger coalitions may reduce the probability of break- down due to popular uprisings/revolutions, but increase the risk of regime-ending coups. Figure 5 shows results for our benchmark re-run on five more specific dependent variables, which include breakdowns due to popular uprisings and breakdowns due to coups, but also three other empirically frequent modes of breakdown: self-coups, incumbent-guided liberalization, and other incumbent-guided regime changes (e.g., a military regime junta institutionalizing a dominant party regime). In line with the theoretical discussion, the largest negative point estimate for coalition size pertains to breakdowns due to popular uprisings, and this coefficient is significant at 5%. Interestingly, we also find that larger coalitions miti- gate chances of “other” incumbent-guided transitions, although these coefficient estimates are somewhat smaller.

In contrast, we find positively signed coalition size coefficients for both coups and self-coups. These coalition size coefficients are sizeable, but do not achieve conventional levels of significance (p-values are .18 and .21 for coups and self-coups, respectively). Despite the lack of clear support for any of these latter relationships, one might still echo the insights from Roessler (2011) and speculate from these non- robust patterns that large coalitions are prone to more mistrustful relationships and empower actors who may conduct coups to institute a new regime under their own leadership. This, in turn, could lead current leaders to purge such coalition members as a preventive measure and consolidate power via self-coups. While larger coalitions are associated with weaker threats from outside the regime through popular uprisings, they are at the very least not associated with weaker threats from within in the form of coups or self-coups.

**Figure 6.**
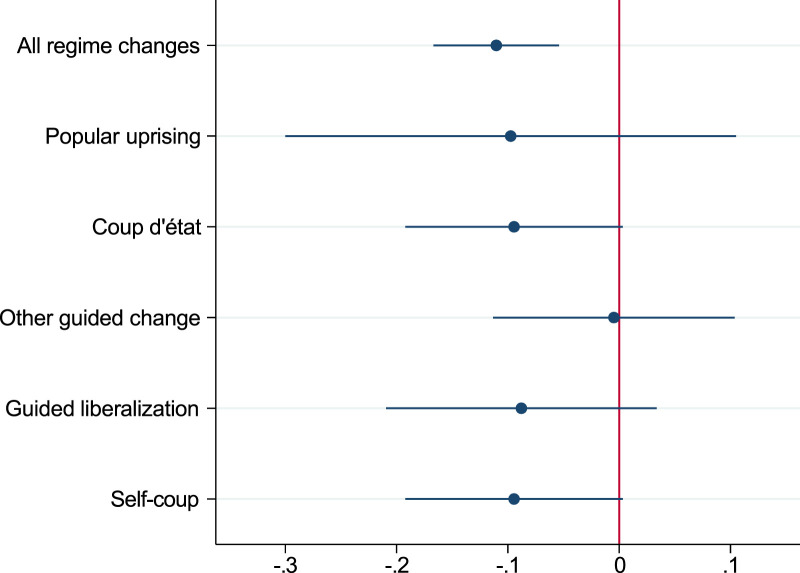
Coefficient estimates for number of support groups. DVs: Types of regime breakdown. Model specifications based on Model 3, Table 1.

Results for coalition diversity are displayed in Figure 6. They shed further light on the fairly robust aggregate finding: A more diverse coalition does not seem to enhance the probability of *any* of the common modes of regime breakdown that we assessed. Whereas coalition size correlates positively with regime-ending coups and self-coups, there is a negative correlation between coalition diversity and these modes of breakdown. Further, more diverse coalitions correspond with lower estimated risks of regime death due to popular uprisings or incumbent-guided liberalization, although the latter results are insignificant. Yet, keeping the caveats on causal identification in mind, the consistency of these results provide at least partial corroboration of the notion that more heterogeneous support coalitions make regimes more resilient against different types of threats.

## Conclusion

We have discussed how different features of regime support coalitions relate to regime survival. First, we discussed expectations, pointing in different directions, concerning how coalition size influences overall probability of regime breakdown, but also expectations on specific modes of regime breakdown. Next, we presented an argument implying that more diverse coalitions – comprising different social groups – lead to more durable regimes. Even when holding coalition size constant, a more diverse coalition should allow regimes to draw on a broader of variety of power resources, which can be used in different scenarios to mitigate different types of threats.

Drawing on recently collected global data, which allow us to capture both coalition size and diversity in a more direct manner than previous large-n studies, we tested our expectations on about 19,500 country-year observations. We find a robust, negative relationship between coalition size and chances of regime breakdown, although sub-sample analysis show that results are most robust in the post-1900 era and in autocratic regimes. More fine-grained analysis indicates that regimes with larger coalitions are far less likely to die from popular uprisings, but perhaps more likely to die from coups or self-coups, although these latter results are not robust. Second, we find a fairly robust relationship between having more diverse regime support coalitions and more durable regimes. We discussed how we cannot guard against all potential sources of confounding; yet, one plausible interpretation of the reported results is the following: Regimes that can draw on the support from various social groups, and thereby a wider variety of power resources, are especially effective in mitigating different threats, both from within and from outside the circle of regime supporters.

Our findings shed new light on some of the long-standing debates on the drivers of political development and (in-)stability. For example, Huntington’s (1968) pioneering book suggests that a key driver of political instability and conflict is the clash between narrow ruling coalitions comprising “traditional” elites, such as the landed aristocracy, and emerging, larger groups empowered by modernization. Our findings that regimes with more sizeable and diverse support coalitions are longer-lived might, in part, reflect that regimes that are willing and able to incorporate such emerging groups in big-tent coalitions can help moderate the potential conflicts highlighted by Huntington. A second example is the debate between “consociationalists”, who emphasize political power-sharing enabled by various formal institutions as an anti-dote to inter-group conflict and related instability in democracies (e.g., Lijphart, 1999), and those who are more skeptical (e.g., Horowitz, 2000). Our study illuminates this debate from a new vantage point by suggesting, more broadly, the potentially regime-stabilizing effects of including different social groups in regime support coalitions in democracies as well as autocracies.

Our findings also exemplify the potential benefits for large-n cross-country studies in political science from moving beyond the (important) role played by institutions, and explicitly considering who the powerful actors in the system are. While we have considered regime survival and breakdown, we surmise that actors characteristics such as coalition size and diversity are likely to, more generally, influence what policies are selected and implemented in a country, and thus have important downstream implications also for other political outcomes, as well as economic and social ones. Hence, we hope that our study can contribute to a future research agenda that investigates the extent to which the social background and heterogeneity of regime support coalitions systematically relate to different outcomes of interest to political scientists, including the pursuit of different social and economic policies as well as decisions on war and peace.

## Supplemental Material

Supplemental Material - Who Rules? Support Coalitions and Regime Survival, 1789–2020 Supplemental Material for Who Rules? Support Coalitions and Regime Survival, 1789–2020 by Carl Henrik Knutsen, Sirianne Dahlum, Magnus Bergli Rasmussen, Tore Wig in Comparative Political Studies

## Data Availability

All replication code and data is available at https://doi.org/10.7910/DV.N/UD7E0E (Knutsen et al., 2025b).

## Notes

1. Replication materials and code can be found at Knutsen et al., (2025b).

2. To illustrate some of this variation in our regime support coalition data, the Qajar Dynasty ruling Persia from 1789–1925 drew on the support from a wide range of groups, including the aristocracy, agrarian elites, and various ethnic and religious groups. The Isabeline regime in Spain (1844–1867) also relied on a heterogeneous coalition comprising the aristocracy, business elites, urban middle classes, party elites, and the military. In contrast, the 1800s Chakri Dynasty in Thailand was supported mainly by the aristocracy and the Rosas-regime in Argentina (1829-52) by the military. Even in democracies, where coalitions are typically larger and broader, there is substantial variation. In present-day New Zealand, for instance, broad population segments – across socio-economic classes and the urban-rural divide – are counted as support groups, but so are various elite groups such as party elites, business elites, state bureaucrats, and the military. Contrast this with Argentina with Isabel Perón as president in 1975, the year before the regime was abruptly ended by a military coup. This relatively democratic regime drew heavily on urban working class support, and our measures only count three support groups, in total.

3. For a discussion of overlaps and differences between key such concepts, see Knutsen et al. (2025a).

4. The selectorate concept has been criticized for being ambiguous and lacking relevance in author itarian settings (e.g. Gallagher & Hanson, 2015), and Bueno de Mesquita and colleagues have also de-emphasized the selectorate’s relevance in later work (Bueno De Mesquita, 2010).

5. Roessler’s (2011) argument linking coalition breadth to coup risk assumes that most coups originate with regime insiders, who are, e.g., privy to information required for a successful coup (leader’s travel schedule, fractions within the military, etc.). The more players in the inner circles of power, the more likely disagreements, fractions, and grievances within these circles become. Moreover, coalitions with many players create what Roessler (2011) calls an “internal security dilemma”, whereby (sub-sets of) elite members suspect others of planning to band together and take power at the others’ expense, thus incentivizing pre-emptive coups.

6. Additionally, institutional adoption and change may be endogenous to the underlying risk of different types of regime breakdown such as coups and revolutions (Bueno de Mesquita & Smith, 2023).

7. One notable exception is the new Political Settlements Dataset (PSD) by Schulz and Kelsall (2021). PSD contains numerous expert-coded variables on, inter alia, the size and composition of the “Leader Bloĉa. There are, however, conceptual (e.g., PSD measuring coalitions behind leaders rather than regimes) and methodological differences between PSD and the measures used in our study, as detailed by Knutsen et al., 2025a. Moreover, the PSD has less extensive coverage, incorporating 42 Global South countries across 1946–2018.

8. Bueno de Mesquita et al. (2003) explicitly theorize that the so-called “loyalty norm” depends on the relative size of the “winning coalition” and “selectorate”. According to their theoretical model, the loyalty norm should be particularly strong when the winning coalition is small and the selectorate is large, since the likelihood for current members of being excluded from future coalitions is especially high.

9. There is, of course, more to coalition diversity than the number of social groups represented. For instance, broad groups such as the urban middle classes may be made up of several sub-groups, such as shopkeepers, academics, and professionals. Moreover, certain groups may be partial substitutes, since they bring fairly similar power resources to the table. Hence, the added effects on regime survival of, e.g., the regime accessing the financial resources of urban business elites may thus be smaller if they already can access the financial resources of large-scale landowners. Our general argument should be read with these caveats in mind.

10. A corresponding single-selection variable asks for *the most important group*.

11. Yet, pairwise correlation coefficients range between .91 and .97 for the 14 different support groups when correlating the original measures using all expert coding with re-calculated measures randomly dropping one expert coder; see Table A8, p. xxxiii in Knutsen et al., 2025a.

12. A drawback is that we ignore a few very short-lived regimes. Yet, these regimes are probably harder even for experts to code and their inclusion would give repeated observations on the annually measured covariates.

13. GDP p.c. growth is calculated from Fariss et al. (2022). The autocratic regime types dummies, which reflect Geddes et al.’s (2014) categories, are constructed from the long time-series regime coding by Anckar and Fredriksson (2019). All other controls were culled from V-Dem, v.12.

14. It is also hard to interpret what it means to increase number of support groups (in the diversity measure) by 1 when holding all support group dummies constant.
