# Supplementary material for: Who Rules? Support Coalitions and Regime Survival, 1789–2020
Source: Comp Polit Stud. 2025 Aug 20;59(9):1932–68. doi: 10.1177/00104140251369338 (PMC13345374; doi:10.1177/00104140251369338)
Supplement: Supplemental Material - Who Rules? Support Coalitions and Regime Survival, 1789–2020 [file sj-pdf-1-cps-10.1177_00104140251369338.pdf]

A Online Appendix

Table of contents

- Appendix A.1: Data on regime support coalition characteristics, pp. i–ii.
- Appendix A.2: Robustness tests, pp. iii–xii.

A.1 Data on regime support coalition characteristics

Below are the expert-coded questions that we use to measure regime support group size and social diversity, the latter defined as the number of social groups that participate in a regime support coalition.

A.1.1 Regime support groups size (v2regsupgroupssize)

Question: In total, how large is the percentage share of the domestic adult (18+) population that belongs to the political regimeâs supporting groups? Do not code for years denoted as âinterregnumâ for the previous, pre-coded question on regime information.
Clarification: You should consider the sum of all the groups (excepting foreign governments and colonial powers) entered in v2regsupgroups. Hence, your answer should take into account the total size of the/those groups that are supportive of the regime, and, if it/they were to retract support would substantially increase the chance that the regime would lose power. Regarding the issue of overlapping identities, and one individual potentially belonging to more than one groups: Individuals should only be "counted" once; thus if the two relevant supporting groups are (4) civil servants, which total about 5 percent, and all of them belong to a particular ethnic group (6) also coded as a relevant, the overall total size of the supporting groups is still 5(presuming that no other members of that ethnic group are essential for the regime staying in power).
Responses:

0: Extremely small (About 1 percent of the population or less; examples of this could include regimes supported by – and needing the support from – a handful of higher-rank military officers, or by only a royal council and a few hundred landowners)

1: Very small (Between 1 percent and 5 percent of the population; examples of this could include regimes supported by – and needing the support from – higher ranking civil servants and the military, or by moderately sized business and agrarian elites)

2: Small (Between 5 percent and 15 percent; examples of this could include regimes supported by – and needing the support from – relatively small ethnic groups, or by urban elites and the urban middle classes in predominantly rural societies)

3: Moderate (Between 15 percent and 30 percent; examples of this could include regimes supported by – and needing the support from – moderately sized ethnic groups, by rural middle classes in rural societies, or by urban middle classes in urban societies)

4: Large (More than 30 percent; examples of this could include regimes supported by – and needing the

support from – large ethnic groups (and then not only the elites/leaders of such groups), or by rural working classes in rural societies.)

Years: 1789-2018

A.1.2 Regime support groups (v2regsupgroups)

Question: Which groups does the current political regime rely on in order to maintain power? (Check all that apply.)

Clarification: Consider which group(s) is supportive of the regime, and, if it/they were to retract support would substantially increase the chance that the regime would lose power. Do not code for years denoted as "interregnum" for the previous, pre-coded question on regime information.

Responses: 0: The aristocracy, including high status hereditary social groups and castes [$v2regsupgroups_0$]

1: Agrarian elites, including rich peasants and large landholders [$v2regsupgroups_1$]

2: Party elites (of the party or parties that control the executive) [$v2regsupgroups_2$]

3: Business elites [$v2regsupgroups_3$]

4: The state bureaucracy [$v2regsupgroups_4$]

5: The military [$v2regsupgroups_5$]

6: An ethnic or racial group(s) [$v2regsupgroups_6$]

7: A religious group(s) [$v2regsupgroups_7$]

8: Local elites, including customary chiefs [$v2regsupgroups_8$]

9: Urban working classes, including labor unions [$v2regsupgroups_9$]

10: Urban middle classes [$v2regsupgroups_1 0$]

11: Rural working classes (e.g. peasants) [$v2regsupgroups_1 1$]

12: Rural middle classes (e.g., family farmers) [$v2regsupgroups_1 2$]

13: A foreign government or colonial power [$v2regsupgroups_1 3$]

Years: 1789-2018

A.2 Robustness tests and additional analyses

Table (A.1) Descriptive statistics for all variables, based on 18,855 observations from Model 1.4

Variable	Mean	Std. dev.	Min.	Max.
Regime breakdown	0.07	0.25	0	1
Regime duration	22.21	31.14	0	293.75
Regime support coalition size	0.27	1.39	-3.55	2.93
Regime support coalition diversity	3.88	2.23	0	11
Ln GDP per capita	8.12	1.05	5.65	11.62
Ln population	15.49	1.70	10.12	21.15
Electoral democracy index	0.31	0.27	0.00	0.93

Table (A.2) Replicating Table 1 and Table 3, but only including independent states

Sample	All regimes				Autocracies	Democracies
Model	1	2	3	4	5	6
Regime support coalition size	-0.311***		-0.265***	-0.222***	-0.242**	-0.353
	(-5.53)		(-4.35)	(-3.36)	(-3.15)	(-1.51)
Regime support coal. diversity		-0.145***	-0.118***	-0.101**	-0.108**	-0.0320
		(-5.10)	(-3.86)	(-3.06)	(-2.80)	(-0.39)
Ln GDP p.c.	-0.389**	-0.508***	-0.405**	-0.398**	-0.249	-0.308
	(-2.82)	(-3.55)	(-2.80)	(-2.62)	(-1.64)	(-0.65)
Ln Population	0.235	0.311*	0.314*	0.330*	0.0953	1.759**
	(1.61)	(2.23)	(2.23)	(2.33)	(0.67)	(2.83)
Electoral democracy index				-0.645+		
				(-1.71)		
Cubic regime duration controls	Y	Y	Y	Y	Y	Y
Country dummies	Y	Y	Y	Y	Y	Y
Year dummies	Y	Y	Y	Y	Y	Y
R^2	0.118	0.118	0.123	0.123	0.111	0.241
ll	-3447.5	-3412.3	-3389.3	-3335.6	-2606.4	-568.4
N	14,811	14,721	14,678	14,419	9,355	3,431

Notes: + $p < .10$; * $p < .05$; ** $p < .01$; *** $p < .001$. T-values in parentheses. Standard errors are clustered on country. The unit of analysis is country-year, and the dependent variable is a dummy for regime breakdown, measured in $t + 1$ (all covariates are measured in t).In models 5 and 6 autocracies and democracies are operationalized as regimes scoring, respectively, 0-3 and 4-6 on Skaaning et al.'s Lexical Index of Electoral Democracy. Independent state is operationalized as countries scoring 1 on V-Dem's v2svindep indicator.

Table (A.3) Replicating Table 1 and Table 3 using OLS instead of Logit

Sample	All regimes				Autocracies	Democracies
Model	1	2	3	4	5	6
Regime support coalition size	-0.0199***		-0.0166***	-0.0150***	-0.0189***	-0.0201*
	(-6.39)		(-4.82)	(-3.82)	(-3.81)	(-2.33)
Regime support coal. diversity		-0.00829***	-0.00577***	-0.00468**	-0.00646*	-0.000779
		(-5.35)	(-3.36)	(-2.61)	(-2.54)	(-0.33)
Ln GDP p.c.	-0.0167**	-0.0184**	-0.0161**	-0.0152*	-0.0122	-0.00401
	(-3.12)	(-3.35)	(-2.92)	(-2.54)	(-1.54)	(-0.30)
Ln Population	-0.000198	0.000191	0.000985	0.000466	0.00380	0.0145
	(-0.03)	(0.03)	(0.16)	(0.08)	(0.39)	(1.18)
Electoral democracy index				-0.0365+		
				(-1.78)		
Cubic regime duration controls	Y	Y	Y	Y	Y	Y
Country dummies	Y	Y	Y	Y	Y	Y
Year dummies	Y	Y	Y	Y	Y	Y
R^2	0.0626	0.0610	0.0636	0.0639	0.0713	0.112
ll	-248.6	-276.4	-236.6	-283.1	-1390.3	2168.4
N	20,207	20,092	19,954	19,457	13,615	6,143

Notes: + $p < .10$; * $p < .05$; ** $p < .01$; *** $p < .001$. T-values in parentheses. Standard errors are clustered on country. The unit of analysis is country-year, and the dependent variable is a dummy for regime breakdown, measured in $t+1$ (all covariates are measured in t).In models 5 and 6 autocracies and democracies are operationalized as regimes scoring, respectively, 0-3 and 4-6 on Skaaning et al.'s Lexical Index of Electoral Democracy.

Table (A.4) Altering the threshold for registering group as support group when calculating diversity measure, for Model 3, Table 1

Threshold for counting as "support group"	Original (0.50)	0.40	0.45	0.55	0.60
Regime support coalition size	-0.214***	-0.209***	-0.213***	-0.231***	-0.242***
	(-4.35)	(-4.19)	(-4.35)	(-4.83)	(-5.07)
Regime support coalition diversity	-0.110***	-0.0895***	-0.110***	-0.103***	-0.0895**
	(-3.84)	(-3.42)	(-3.80)	(-3.87)	(-2.97)
Ln GDP p.c.	-0.475***	-0.486***	-0.474***	-0.466***	-0.475***
	(-4.33)	(-4.42)	(-4.31)	(-4.22)	(-4.33)
Ln Population	0.173	0.179	0.174	0.153	0.153
	(1.46)	(1.49)	(1.46)	(1.25)	(1.26)
Cubic regime duration controls	Y	Y	Y	Y	Y
Country dummies	Y	Y	Y	Y	Y
Year dummies	Y	Y	Y	Y	Y
R^2 (pseudo)	0.119	0.118	0.119	0.118	0.117
ll	-4358.5	-4362.8	-4358.8	-4362.7	-4365.2
N	19,256	19,256	19,256	19,256	19,256

Notes: + $p < .10$; * $p < .05$; ** $p < .01$; *** $p < .001$. Coefficients from logistic regressions with t-values in parentheses. Standard errors are clustered on country. The unit of analysis is country-year, and the dependent variable is a dummy for regime breakdown, measured in $t+1$ (all covariates are measured in t)

Table (A.5) Omitting covariates

Model	1	2	3	4	5	6
Regime support coalition size	-0.196***	-0.163***	-0.154***	-0.0700*	-0.0916***	
	(-6.52)	(-4.28)	(-4.36)	(-2.39)	(-3.60)	
Regime support coal. diversity	-0.0758***	-0.0864***	-0.0855***	-0.0406+		-0.0584**
	(-3.82)	(-3.37)	(-3.52)	(-1.89)		(-3.01)
Ln GDP p.c.	-0.171***	-0.244***	-0.249***			
	(-3.87)	(-4.95)	(-5.10)			
Ln Population	0.00661	-0.00205				
	(0.31)	(-0.07)				
R^2 (pseudo)	0.0521	0.0301	0.0286	0.00326	0.00247	0.00205
ll	-4736.5	-4845.7	-5073.3	-6333.7	-6424.6	-6382.6
N	19,954	19,966	21,007	25,552	25,920	25,691

Notes: + $p < .10$; * $p < .05$; ** $p < .01$; *** $p < .001$. T-values in parentheses. Standard errors are clustered on country. The unit of analysis is country-year, and the dependent variable is a dummy for regime breakdown, measured in $t + 1$ (all covariates are measured in t).

Table (A.6) Adding extra controls, one (set) at the time, to Model 3, Table 1

Model	1	2	3	4	5	6
Support coalition size	-0.221***	-0.306***	-0.229***	-0.202***	-0.178***	-0.258***
	(-4.49)	(-3.95)	(-4.37)	(-4.11)	(-3.60)	(-4.01)
Support coal. diversity	-0.105***	-0.189***	-0.117***	-0.120***	-0.109***	-0.125***
	(-3.66)	(-4.17)	(-3.59)	(-3.97)	(-3.84)	(-3.99)
Ln GDP p.c.	-0.423***	-0.606**	-0.544***	-0.483***	-0.355**	-0.449**
	(-3.84)	(-2.61)	(-5.02)	(-4.65)	(-3.25)	(-2.92)
Ln Population	0.133	0.674**	0.0588	0.111	0.198+	0.435**
	(1.13)	(2.68)	(0.47)	(0.96)	(1.84)	(3.04)
GDP p.c. growth	-0.0513***					
	(-4.44)					
Ln nat. res. income p.c.		-0.0336				
		(-0.56)				
Urbanization			-0.195			
			(-0.48)			
International conflict				0.275*		
				(2.42)		
Internal conflict				0.293*		
				(2.40)		
Impartial administration					-0.201***	
					(-4.43)	
Dominant party regime						-0.290
						(-1.47)
Personalist regime						0.429**
						(3.05)
Military regime						0.216
						(1.41)
Autocratic monarchy						-0.124
						(-0.59)
Cubic regime duration controls	Y	Y	Y	Y	Y	Y
Country dummies	Y	Y	Y	Y	Y	Y
Year dummies	Y	Y	Y	Y	Y	Y
R^2 (pseudo)	0.122	0.126	0.117	0.117	0.122	0.129
ll	-4318.3	-2211.3	-3466.8	-3866.7	-4319.2	-3250.6
N	19,079	9,293	14,448	16,113	19,090	14,172

Notes: + $p < .10$; * $p < .05$; ** $p < .01$; *** $p < .001$. Coefficients from logistic regressions with t-values in parentheses. Standard errors are clustered on country. The unit of analysis is country-year, and the dependent variable is a dummy for regime breakdown, measured in $t + 1$ (all covariates are measured in t)

Table (A.7) Replicating Model 3, Table 1, but controlling for particular group being included in the support coalition.

Model	1	2	3	4	5	6	7	8	9	10	11	12	13
Support coalition size	-0.214***	-0.202***	-0.222***	-0.216***	-0.214***	-0.209***	-0.218***	-0.215***	-0.214***	-0.207***	-0.202***	-0.199***	-0.214***
	(-3.98)	(-4.17)	(-4.23)	(-4.30)	(-4.37)	(-4.22)	(-4.43)	(-4.38)	(-4.39)	(-4.03)	(-4.03)	(-3.99)	(-4.36)
Support coal. diversity	-0.110***	-0.133***	-0.116***	-0.0945**	-0.0884**	-0.125***	-0.125***	-0.102***	-0.109***	-0.102**	-0.0953**	-0.1000**	-0.111***
	(-3.41)	(-4.27)	(-3.77)	(-2.78)	(-2.92)	(-4.22)	(-3.92)	(-3.67)	(-3.75)	(-3.25)	(-3.15)	(-3.15)	(-3.69)
Ln GDP p.c.	-0.475***	-0.457***	-0.471***	-0.457***	-0.480***	-0.450***	-0.477***	-0.480***	-0.475***	-0.464***	-0.455***	-0.463***	-0.476***
	(-4.33)	(-4.11)	(-4.26)	(-4.28)	(-4.34)	(-4.20)	(-4.33)	(-4.36)	(-4.32)	(-4.08)	(-4.03)	(-4.29)	(-4.32)
Ln Population	0.173	0.149	0.176	0.190	0.166	0.149	0.175	0.182	0.174	0.156	0.159	0.142	0.175
	(1.44)	(1.21)	(1.48)	(1.58)	(1.42)	(1.24)	(1.47)	(1.51)	(1.46)	(1.29)	(1.34)	(1.12)	(1.43)
The Aristocracy	-0.00363												
	(-0.02)												
Agrarian elites		0.249*											
		(2.00)											
Party elites			0.0686										
			(0.60)										
Business elites				-0.141									
				(-1.06)									
State bureaucracy					-0.190+								
					(-1.84)								
The military						0.170+							
						(1.65)							
Ethnic/racial group							0.227						
							(1.11)						
Religious group								-0.154					
								(-0.75)					
Local elites									-0.0114				
									(-0.09)				
Urban working classes										-0.177			
										(-0.80)			
Urban middle classes											-0.257		
											(-1.37)		
Rural working classes												-0.291	
												(-1.40)	
Rural middle classes													0.0155
													(0.09)
r2													
p	0.119	0.119	0.119	0.119	0.119	0.119	0.119	0.119	0.119	0.119	0.119	0.119	0.119
ll	-4358.5	-4355.1	-4358.2	-4357.4	-4356.4	-4356.3	-4356.9	-4357.9	-4358.5	-4357.5	-4355.9	-4356.3	-4358.5
N	19256	19256	19256	19256	19256	19256	19256	19256	19256	19256	19256	19256	19256
R2 (pseudo)	0.0397	0.0396	0.0389	0.0392	0.0390	0.0391	0.0394	0.0393	0.0390	0.0389	0.0391	0.0390	0.0389
ll	-4105.9	-4106.5	-4109.3	-4108.2	-4108.9	-4108.7	-4107.2	-4107.7	-4108.9	-4109.2	-4108.5	-4109.1	-4109.3
N	17223	17223	17223	17223	17223	17223	17223	17223	17223	17223	17223	17223	17223
Duration dummies?	yes	yes	yes	yes	yes	yes	yes	yes	yes	yes	yes	yes	yes
Country dummies?	yes	yes	yes	yes	yes	yes	yes	yes	yes	yes	yes	yes	yes
Year dummies?	yes	yes	yes	yes	yes	yes	yes	yes	yes	yes	yes	yes	yes

Notes: + $p < .10$; * $p < .05$; ** $p < .01$; *** $p < .001$. Coefficients from logistic regressions with t-values in parentheses. Standard errors are clustered on country. The unit of analysis is country-year, and the dependent variable is a dummy for regime breakdown, measured in $t + 1$ (all covariates are measured in t).

Table (A.8) Replicating Table 1, by time period

Sample	1789-1899				1900-2020			
Model	1	2	3	4	1	2	3	4
Regime support coalition size	-0.228*		-0.181*	-0.131	-0.371***		-0.302***	-0.276***
	(-2.49)		(-2.01)	(-1.34)	(-6.63)		(-5.00)	(-4.25)
Regime support coal. diversity		-0.113+	-0.0915	-0.0884		-0.183***	-0.125**	-0.119**
		(-1.92)	(-1.50)	(-1.45)		(-4.67)	(-3.00)	(-2.75)
Ln GDP p.c.	-0.543	-0.499	-0.505	-0.263	-0.493***	-0.496**	-0.481**	-0.455**
	(-1.09)	(-1.14)	(-1.01)	(-0.39)	(-3.35)	(-3.22)	(-3.16)	(-2.94)
Ln Population	-0.152	-0.0922	-0.114	-0.0920	0.796***	0.761***	0.839***	0.809***
	(-0.53)	(-0.30)	(-0.38)	(-0.32)	(3.77)	(3.59)	(3.99)	(3.84)
Electoral democracy index				-1.823				-0.362
				(-1.21)				(-0.99)
Cubic regime duration controls	Y	Y	Y	Y	Y	Y	Y	Y
Country dummies	Y	Y	Y	Y	Y	Y	Y	Y
Year dummies	Y	Y	Y	Y	Y	Y	Y	Y
R^2 (pseudo)	0.138	0.134	0.138	0.132	0.126	0.124	0.129	0.129
ll	-1154.8	-1143.6	-1104.0	-1052.1	-3179.7	-3186.2	-3167.0	-3143.5
N	4,886	4,802	4,642	4,346	14,244	14,242	14,242	14,181

Notes: + $p < .10$; * $p < .05$; ** $p < .01$; *** $p < .001$. Coefficients from logistic regressions with t-values in parentheses. Standard errors are clustered on country. The unit of analysis is country-year, and the dependent variable is a dummy for regime breakdown, measured in $t + 1$ (all covariates are measured in t).

Table (A.9) Replicating Table 1, across 1946–2010, for, respectively Djuve et al (2020) and Geddes et al. (2014) measures of regime breakdown and regime duration.

Data source regime duration/breakdown	DV: Djuve et al.				DV: Geddes et al.			
Model	1	2	3	4	1	2	3	4
Regime support group size	-0.349***		-0.291***	-0.292***	-0.379*		-0.297*	-0.290
	(-4.50)		(-3.65)	(-3.48)	(-2.49)		(-2.00)	(-1.59)
Regime support coal. diversity		-0.190***	-0.129*	-0.129*		-0.260**	-0.216*	-0.215*
		(-3.32)	(-2.22)	(-2.22)		(-2.83)	(-2.35)	(-2.39)
Ln GDP p.c.	-0.499*	-0.451*	-0.474*	-0.475*	-0.804*	-0.877*	-0.767*	-0.763*
	(-2.51)	(-2.27)	(-2.35)	(-2.30)	(-2.25)	(-2.38)	(-2.07)	(-2.02)
Ln Population	0.0823	0.0613	0.122	0.123	-1.655*	-1.603*	-1.544*	-1.553*
	(0.24)	(0.18)	(0.35)	(0.34)	(-2.15)	(-2.11)	(-2.03)	(-1.97)
Electoral democracy index				0.0150				-0.0656
				(0.03)				(-0.07)
Cubic regime duration controls	Y	Y	Y	Y	Y	Y	Y	Y
Country dummies	Y	Y	Y	Y	Y	Y	Y	Y
Year dummies	Y	Y	Y	Y	Y	Y	Y	Y
R^2 (pseudo)	0.116	0.113	0.118	0.118	0.135	0.136	0.139	0.139
ll	-1942.3	-1948.8	-1937.6	-1937.6	-1006.7	-1005.4	-1001.2	-1001.2
N	8,265	8,265	8,265	8,265	4,894	4,894	4,894	4,894

Notes: + $p < .10$; * $p < .05$; ** $p < .01$; *** $p < .001$. Coefficients from logistic regressions with t-values in parentheses. Standard errors are clustered on country. The unit of analysis is country-year, and the dependent variable is a dummy for regime breakdown, measured in $t + 1$ (all covariates are measured in t).

Table (A.10) Replicating Models 1,2 and 4, Table 1 on regime-specific sub-samples

Sample	Only autocracies			Only democracies		
Model	1	2	3	4	5	6
Regime support coalition size	-0.261***		-0.312***	-0.363		0.0931
	(-4.57)		(-4.80)	(-1.64)		(0.38)
Regime support coal. diversity		-0.125***	-0.0983**		-0.101	-0.0434
		(-3.86)	(-2.63)		(-1.20)	(-0.57)
Ln GDP p.c.	-0.209	-0.296*	-0.278+	-0.429	-0.396	0.132
	(-1.63)	(-2.36)	(-1.95)	(-0.99)	(-0.89)	(0.29)
Ln Population	-0.0334	0.0389	-0.0312	1.243*	1.134*	1.671**
	(-0.26)	(0.29)	(-0.21)	(2.47)	(2.13)	(3.05)
Electoral democracy index			3.304***			-6.461***
			(5.07)			(-7.32)
Cubic regime duration controls	Y	Y	Y	Y	Y	Y
Country dummies	Y	Y	Y	Y	Y	Y
Year dummies	Y	Y	Y	Y	Y	Y
R^2 (pseudo)	0.110	0.108	0.116	0.231	0.229	0.262
ll	-3515.2	-3503.5	-3354.0	-617.9	-618.9	-590.1
N	13,258	13,172	12,640	3,845	3,845	3,836

Notes: + $p < .10$; * $p < .05$; ** $p < .01$; *** $p < .001$. Coefficients from logistic regressions with t-values in parentheses. Standard errors are clustered on country. The unit of analysis is country-year, and the dependent variable is a dummy for regime breakdown, measured in $t + 1$ (all covariates are measured in t).In models 5 and 6 autocracies and democracies are operationalized as regimes scoring, respectively, 0-3 and 4-6 on Skaaning et al.'s Lexical Index of Electoral Democracy.

Table (A.11) Replicating Table 2 on sub-sample of autocratic regimes (< 4 on LIED)

Type test	OLS	No country-FE	No year-FE	Adding controls	Control group id.	3-year lag	10-year lag
Model	1	2	3	4	5	6	7
Support coalition size	-0.0189***	-0.144***	-0.216***	-0.254*	-0.191**	-0.298***	-0.192*
	(-3.81)	(-3.69)	(-4.02)	(-2.16)	(-3.15)	(-4.52)	(-2.48)
Support coal. diversity	-0.00646*	-0.0412+	-0.115***	-0.244***	0.138	-0.0619+	-0.0490
	(-2.54)	(-1.90)	(-3.71)	(-3.80)	(0.86)	(-1.75)	(-1.41)
Ln GDP p.c.	-0.0122	-0.0643	-0.164	-0.433	-0.183	-0.117	-0.193
	(-1.54)	(-1.25)	(-1.49)	(-1.48)	(-1.51)	(-0.92)	(-1.28)
Ln Population	0.00380	-0.00874	0.287**	-0.341	-0.0139	-0.0135	-0.139
	(0.39)	(-0.39)	(3.18)	(-0.96)	(-0.09)	(-0.09)	(-0.86)
GDP p.c. growth				-0.0707***			
				(-4.12)			
Ln nat. res. income p.c.				-0.0338			
				(-0.36)			
Urbanization				-0.307			
				(-0.25)			
International conflict				0.104			
				(0.45)			
Internal conflict				0.0731			
				(0.40)			
Impartial administration				0.0385			
				(0.33)			
Dominant party regime				-0.647+			
				(-1.95)			
Personalist regime				0.310			
				(1.19)			
Military regime				-0.0323			
				(-0.12)			
Autocratic monarchy				0.366			
				(0.92)			
State bureaucracy					-0.467*		
					(-2.40)		
Religious group					-0.845**		
					(-2.72)		
Rural working classes					-0.979**		
					(-2.77)		
Cubic regime duration	Y	Y	Y	Y	Y	Y	Y
Country dummies		Y	Y	Y	Y	Y	Y
Year dummies	Y		Y	Y	Y	Y	Y
R^2 (pseudo)		0.0778	0.0659	0.135	0.119	0.142	0.139
ll	-1390.3	-3615.9	-3650.9	-1348.2	-3426.0	-3388.7	-3241.4
N	13,615	13,380	13,241	4,796	13,015	13,130	12,757

Notes: + $p < .10$; * $p < .05$; ** $p < .01$; *** $p < .001$. T-values in parentheses. Standard errors are clustered on country. The unit of analysis is country-year. Dummies for all 14 support groups are entered in Model 4, but coefficients for all such dummies with p> 0.05 are omitted to shrink the table.

Figure (A.1) Only autocratic regimes: Predicted probabilities of regime breakdown, for different sizes of regime support coalitions (panel a) and different levels of coalition diversity (panel b). Benchmark estimated on subset of observations (only autocracies)

(a) a (b) b

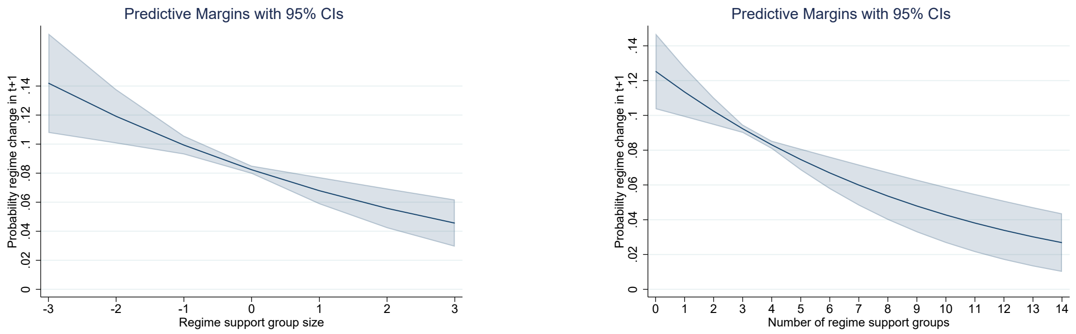

Predictions are from the benchmark models re-run only on autocratic regime observations (Model 5, Table 1), and made for observations that have mean scores on all covariates. Autocracies are defined as regimes that lack competitive multi-party elections, scoring 0-3 on Skaaning et al.'s Lexical Index of Electoral Democracy.

Figure (A.2) Assessing non-monotonic relationships: Predicted probabilities of regime breakdown, across size (left panel) or heterogeneity (right panel) of regime support coalitions, controlling also for democracy.

(a) a (b) b

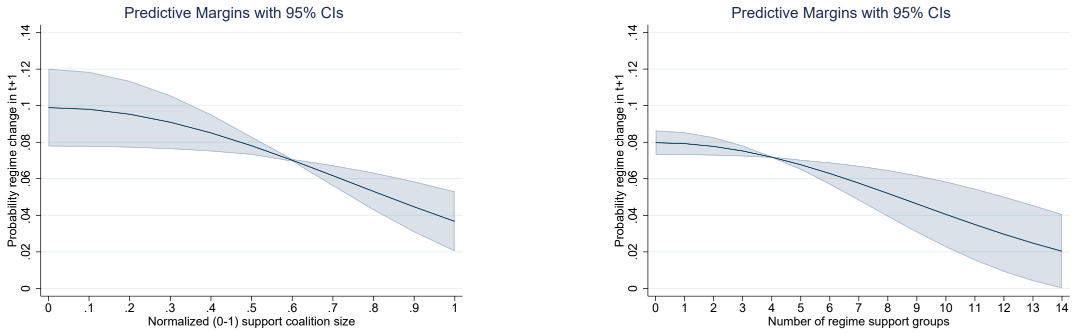

Predictions are from a version of Model 4, Table 1 including squared terms on support coalition size, support coalition diversity and electoral democracy index (panel b). All covariates at their means.

Figure (A.3) Assessing non-monotonic relationships: Predicted probabilities of regime breakdown, for different sizes of regime support coalitions. Linear probability (OLS) models

(a) a (b) b

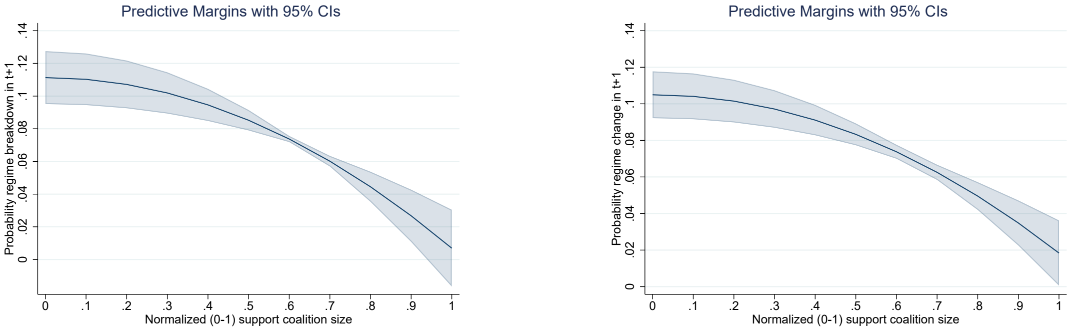

Predictions are from an OLS version of Model 3, Table 1 including squared terms on support coalition size and support coalition diversity (panel a) and an OLS version of Model 4, Table 1 including squared terms on support coalition size, support coalition diversity and electoral democracy index (panel b). Predictions are made for observations that have mean scores on all covariates except support coalition size.

Figure (A.4) Assessing non-monotonic relationships: Predicted probabilities of regime breakdown, for different numbers of regime support groups. Linear probability (OLS) models

(a) a (b) b

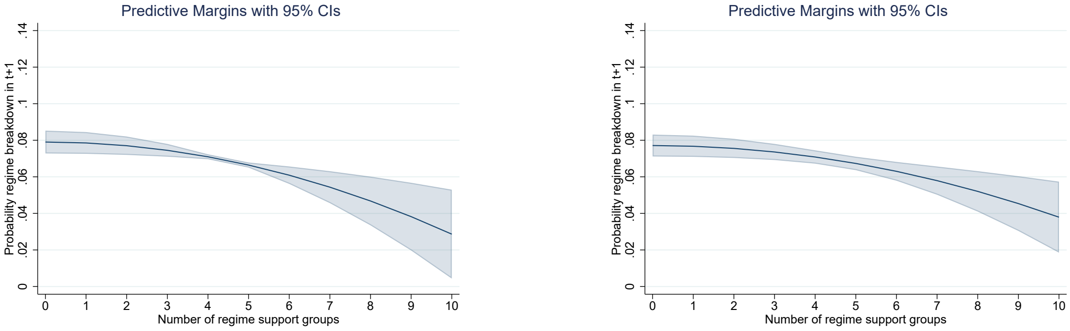

Predictions are from an OLS version of Model 3, Table 1 including squared terms on support coalition size and support coalition diversity (panel a) and an OLS version of Model 4, Table 1 including squared terms on support coalition size, support coalition diversity and electoral democracy index (panel b). Predictions are made for observations that have mean scores on all covariates except support coalition diversity as measured by number of support groups. In the lower plot, clustered standard errors could not be produced, so 95% CIs are based on classical errors.
